# Supplementary material for: Person-centred care transitions for people with stroke: study protocol for a feasibility evaluation of codesigned care transition support
Source: BMJ Open. 2021 Dec 22;11(12):e047329. doi: 10.1136/bmjopen-2020-047329 (PMC8710855; doi:10.1136/bmjopen-2020-047329)
Supplement: Author's manuscript [file bmjopen-2020-047329.draft_revisions.pdf]

BMJ Open is committed to open peer review. As part of this commitment we make the peer review history of every article we publish publicly available.

When an article is published we post the peer reviewers' comments and the authors' responses online. We also post the versions of the paper that were used during peer review. These are the versions that the peer review comments apply to.

The versions of the paper that follow are the versions that were submitted during the peer review process. They are not the versions of record or the final published versions. They should not be cited or distributed as the published version of this manuscript.

BMJ Open is an open access journal and the full, final, typeset and author-corrected version of record of the manuscript is available on our site with no access controls, subscription charges or pay-per-view fees (http://bmjopen.bmj.com).

If you have any questions on BMJ Open's open peer review process please email info.bmjopen@bmj.com

BMJ Open

Person-centred care transitions for people with stroke: study protocol for evaluation of feasibility of the co-designed rehabilitation link

Journal:	*BMJ Open*
Manuscript ID	bmjopen-2020-047329
Article Type:	Protocol
Date Submitted by the Author:	30-Nov-2020
Complete List of Authors:	Flink, Maria; Karolinska Institutet, Neurobiology, Care Sciences and Society; Karolinska University Hospital<br>Lindblom, Sebastian; Karolinska Institutet, Neurobiology, Care Sciences and Society; Karolinska University Hospital<br>Tistad, Malin; Dalarna University<br>Laska, Ann Charlotte ; Karolinska Institutet, Department of Clinical Sciences Danderyd Hospital<br>Bertilsson, Bo Christer; Karolinska Institutet, Neurobiology, Care Sciences and Society<br>Wärlinge, Carmen; Region Stockholm, Academic Primary Health Care Centre<br>Hasselström, Jan; Karolinska Institutet, 6. Department of Neurobiology, Care Sciences and Society; Region Stockholm, Academic Primary Health Care Centre<br>Elf, Marie; Dalarna University, School of education, health and social studies<br>von Koch, Lena; Karolinska Institutet, Neurobiology, Care Sciences and Society; Karolinska University Hospital<br>Ytterberg, Charlotte; Karolinska Institutet, Neurobiology, Care Sciences and Society; Karolinska University Hospital
**Primary Subject Heading**:	
Secondary Subject Heading:	
Keywords:	Stroke < NEUROLOGY, REHABILITATION MEDICINE, Organisation of health services < HEALTH SERVICES ADMINISTRATION & MANAGEMENT

SCHOLARONE™
Manuscripts

Person-centred care transitions for people with stroke: study protocol for evaluation of feasibility of the co-designed rehabilitation link

Maria Flink[1,2], Sebastian Lindblom[1,2], Malin Tistad[1,3], Ann Charlotte Laska[4], Bo Christer Bertilson[1], Carmen Wärlinge[5], Jan Hasselström[1,5], Marie Elf[3], Lena von Koch[1,2], Charlotte Ytterberg[1,2]

1. Karolinska Institutet, Department of Neurobiology, Care Sciences and Society, Huddinge, Sweden
2. Karolinska University Hospital, Stockholm, Sweden
3. Dalarna University, School of Education, Health and Social Studies, Falun, Sweden
4. Karolinska Institutet, Department of Clinical Sciences Danderyd Hospital, Stockholm, Sweden
5. Academic Primary Health Care Centre, Stockholm Health Care Services (SLSO), Region Stockholm, Sweden

Corresponding author:

Maria Flink

Alfred Nobels allé 23, D2

Karolinska Institutet

SE- 14183 Huddinge, SWEDEN

maria.flink@ki.se

Word count: 3111

For peer review only

For peer review only - http://bmjopen.bmj.com/site/about/guidelines.xhtml

ABSTRACT

Introduction: Extensive evidence shows that transitions from hospital to home should be bridged through collaboration between professionals in hospitals and subsequent rehabilitation teams, and that well-coordinated transitions can reduce death and disability after a stroke. Despite this, there is no consensus on core components for such transitions, and health services are delivered within organizational structures rather than based on patients' needs. The primary aim is to assess the feasibility operationalized as fidelity, acceptability, and the secondary aim is to assess likely effectiveness of a co-designed rehabilitation link for persons with stroke.

Methods and analysis: This study protocol describes the evaluation of a feasibility study using a non-randomized controlled design. The co-designed rehabilitation link includes patient information using videos, leaflets and teach back; what-matters-to me dialogue; coordinated rehabilitation plan; bridging e-meeting; and a message system for cross-organizational collaboration. The co-designed rehabilitation link will be implemented at a geriatric stroke unit and an acute stroke unit. Data will be collected on the primary outcome satisfaction with the co-designed rehabilitation link, on the secondary outcome health literacy and medication adherence; and use of healthcare. Data on patient outcomes will be compared with matched controls from another geriatric stroke unit and acute stroke unit, and with matched historic controls from a previous dataset at the units in the intervention and control units. Data on acceptability and fidelity will be assessed through interviews and observations at the intervention units. Qualitative data will be analyzed using manifest qualitative content analysis and data on likely effectiveness will be analyzed with test statistics

Ethics and dissemination: The study has ethical approval from the Swedish Ethical Review Authority. The results will be published open-access in peer-reviewed journals.Trial registration: http://www.clinicaltrials.gov id: NCT02925871. Date of registration October 6.

Trial registration: http://www.clinicaltrials.gov id: NCT02925871. Date of registration October 6, 2016

Protocol version: 1

or peer review only

Key words

Stroke, care planning, continuity of patient care, implementation, participation, patient satisfaction, rehabilitation, self-efficacy, significant others, transitional care, user involvement

Strengths and limitations of this study

- The intervention was developed following the guidelines of the Medical Research Council
- The development of the intervention was conducted using co-design methodology with people with stroke, significant other and health care professionals
- The study will use mixed methods to evaluate the feasibility of the trial
- The study is non-randomised with matched and historic controls

For peer review only - http://bmjopen.bmj.com/site/about/guidelines.xhtml

BACKGROUND

Stroke, one of the leading causes of disability[1], has an abrupt onset and is a stressful event, both for the person with stroke, and the family[2]. The past decades have seen improvements in medical and acute care of stroke as well as a reduction of the mean length of hospital stay in stroke units in high income countries[3]. Strong evidence and consensus have shown that stroke care should initially be supplied in stroke units in hospital in the acute phase[4], followed by a period of rehabilitation to regain functioning and to receive psycho-social support[2]. Hence, a care transition – a shift in responsibility from one healthcare setting to another –is almost always needed. This may entail a care transition to inpatient rehabilitation or directly to rehabilitation in the home provided by primary healthcare, e.g. by a multi-professional team.

Uncoordinated care transitions impose a burden to patients and their significant others, especially when they lack information to navigate the healthcare system[2]. Cognitive impairments, post-stroke fatigue and depression[5], as well as the sudden onset, often render the person having had the stroke and their significant others unprepared for the care transition to home[6, 7]. In addition, a short hospital stay, little time to participate in transition planning[8, 9], and a fragmented healthcare may contribute to unrealistic expectations[7] and a sense of being abandoned[10] in a new and complex life situation. Although laws and other regulations require care providers to coordinate care, a great responsibility is put on the patient and significant others to coordinate their own care[11], while few arrangements are offered to support a patient's ability to participate in their own care or to self-manage[12, 13].

For peer review only - http://bmjopen.bmj.com/site/about/guidelines.xhtml

Several care transition interventions have been developed, both for people with stroke and for other populations. Strong evidence supports that to reduce re-hospitalizations, such care transitions must include both pre- and post-discharge activities and support patients' capacity for self-management[14, 15]. The pre- and post-discharge activities include discharge planning, structured discharge information, coordinated follow-up and timely communication between care providers[16, 17]. However, these interventions are not specifically directed to care transitions within rehabilitation or for people with stroke whose capacity for self-management may be hindered by the cognitive and communicative impairments of the stroke. For people with stroke, extensive evidence shows that the rehabilitation care transition Early Supported Discharge (ESD) can reduce death and disability after a mild to moderate stroke[18]. ESD implies that a stroke specific, multidisciplinary team plans and coordinates the hospital discharge, and provides rehabilitation in the community[19]. Despite the evidence, ESD has scarcely been implemented in Sweden. A possible explanation for the lack of implementation is that cross-organizational health services such as ESD may be difficult to accomplish in fragmented healthcare systems with firm borders between hospital and community care.

There is hence a need for a rehabilitation care transition that both takes the patient's needs and capacity, and the organizational settings into consideration. This need was highlighted in our prospective observational study of 206 people with acute stroke, of the current state of transitions from stroke and geriatric hospital units to the home with subsequent rehabilitation in primary healthcare in Stockholm, Sweden[20, 21]. One week after hospital discharge the participants were followed-up regarding satisfaction with care transition and at three- and 12-months regarding functioning in everyday life and use of healthcare. In addition, we performed a grounded theory study of perceptions of current care transitions of patients,

For peer review only - http://bmjopen.bmj.com/site/about/guidelines.xhtml

families, and healthcare professionals. The findings from the pre-studies[20, 21] identified a need for improved dialogue between patients and professionals; between professionals working in the same care provider organization; and between professionals in different organizations. The improved dialogue includes a communication where all parties are involved on equal terms and strive for a common understanding. Further, a need to enhance patients' preparedness for discharge, and for their self-management at home was identified. Based on the results[20, 21] we identified a need to develop bridging communicative links along the transition process, within and between care provider organizations and between patients and healthcare professionals. This aims to ensure a coordinated rehabilitation transition, and patients and significant others to feel prepared for the home coming.

Development of the intervention

The intervention is hence based both on our pre-studies[20, 21] and on literature on the stroke care trajectory[4, 18], and was developed in a co-design process in two steps[22].

First, five co-design workshops led by a trained facilitator were held with people with stroke, a significant other, and healthcare professionals from hospital stroke units and multidisciplinary home rehabilitation teams. The participants identified several unmet needs of patients, significant others, and professionals within three areas of the care transition:

1) Shared understanding of patient illness and situation: repeated information about the present situation and expected future; communication adapted to the situation; updated written information; time to ask questions; structured and timely information.

2) Preparedness for home-coming: feeling safe and understood in the discharge; clarity about services provided by the home rehabilitation team; being prepared for the home-coming; clarity about medications; own perspective and needs taken into consideration.

For peer review only - http://bmjopen.bmj.com/site/about/guidelines.xhtml

3) Coordination: coordination between stroke unit and home rehabilitation teams; clarity about the care trajectory; collaboration between sites.

Based on these needs, suggestions for improvements were co-designed by the participants.

Thereafter, interactive e-meetings with healthcare professionals were held in which suggestions from the co-design workshops were further developed and contextually adapted into the intervention.

The intervention – the co-designed rehabilitation link

The intervention consists of several elements that aim to meet patients', significant others' and professionals' need of shared understanding, patient preparedness for home-coming, and coordination (Figure 1).

Please insert Figure 1 here.

Three different sources of information of home-rehabilitation will be offered to patients and significant others: *two information videos and one leaflet,* before a *what matters to me-dialogue* at the hospital. First, an information video with general information of home-rehabilitation will be shown during daytime in the day room. Second, an information video with the professionals in the patient's specific home-rehabilitation team will be provided to patients on tablets. Third, an information leaflet with general information about home-rehabilitation, a link to the information video, and contact information to the specific team. After the patient has accessed these sources of information the professional will initiate a dialogue between the patient and the professional of "*what matters to me*" in relation to coming home with continued rehabilitation. In this dialogue the professional and the patient will initate the development of a *coordinated rehabilitation plan*, based on the patient's needs and wishes for the continuing rehabilitation at home, as well as on the professionals'

For peer review only - http://bmjopen.bmj.com/site/about/guidelines.xhtml

assessment. Based on agreements in the initiated plan, the professional will write a referral to the home-rehabilitation team and book an e-meeting with the team before discharge. This *bridging e-meeting* aims to connect the patient with the team that will be responsible for rehabilitation at home through a first personal contact and decide an appointment for the first home-visit. The e-meetings will include a dialogue between patient, the significant other, and the hospital professionals and rehabilitation team. The dialogue will include the patient's situation, needs, wishes and fears in relation to coming home; a presentation from the home rehabilitation team of their services; a possibility for the patient and significant other to ask questions; and to continue the initiated coordinated rehabilitation plan for the homecoming. The e-meeting will function as an in-reaching bridge between hospital and the home-rehabilitation team. Thereto, the hospital professionals will add contact information, i.e. the telephone number to the home rehabilitation team in the patient's cell phone, and make sure that the correct contact information to the patient and significant other is available to the home rehabilitation team.

Teach back[23, 24] is a person-centred, iterative communication method focused on shared understanding of the patient's situation and of the professional's information. The aim of *Teach back* is both to ensure that health professionals have understood the patient correctly and an improved patient learning and understanding of the own health condition, medications, and how to navigate the healthcare system, i.e. improved health literacy. Teach-back will be used in the *"what matters to me"*-dialogue, the bridging e-meeting, and discharge encounters. The professionals will give information and thereafter ask the patient to tell in own words what was said, emphasizing that this is to check how the professional explained.

ew only

A safe *instant messaging system* within the electronical health record will ensure inter-organisational communication between healthcare professionals. This will ensure exchange of information and resolve remaining questions on the patient's care between the hospital and home-rehabilitation, for example regarding revoked driving license or medications.

The co-designed rehabilitation link is grounded in principals of person-centred care[25] and of integrated care[26] on a micro and meso level. This entails that the development of the intervention and the actual intervention both are based on the individual's preferences, needs, and values, taking patient resources into consideration, i.e. is person-centred[25], and strives to be realistic regarding what can be provided by the healthcare professionals in the involved organisational settings. We adhere to the person-centred Rainbow model of integrated care[26], emphasizing the need for both functional and normative links between patients and professionals, as well as within and between organisational settings. Functional here refers to the information system that links the different parts and normative to shared understanding and values, both within and between organizations.

Aims and hypothesis

The primary aim is to assess the feasibility operationalized as fidelity, acceptability; and the secondary aim is to assess likely effectiveness of a co-designed rehabilitation link for persons with stroke.

The following research questions (RQ) will be used:

RQ1: Is the intervention delivered as intended (fidelity)?

RQ2: Is the intervention acceptable regarding content and delivery?

For peer review only - http://bmjopen.bmj.com/site/about/guidelines.xhtml

RQ3: Are there any differences in patient and significant other outcomes between patients who received the intervention and matched controls, and between intervention patients and matched historic controls?

We hypothesise that information through a person-centred dialogue with focus on shared understanding between patients and healthcare professionals and a discharge focused on bridging the hospitalization with the home through meetings and coordinated rehabilitation plan will help patients and significant others feel more satisfied with the transition, and be better prepared for the home-coming including the subsequent rehabilitation activities. This may in turn help patients regain functioning and reduce healthcare utilization.

METHODS

This study protocol describes the evaluation of feasibility for the co-designed rehabilitation link using a non-randomized controlled design. Up-dates on the study protocol will be published on Clinical Trials.

The project adheres to the framework for development and evaluation of complex interventions by the Medical Research Council, UK[27], which calls for phased and iterative approaches in the design and evaluation of a complex intervention. This project hence draws upon our pre-studies of the perceptions, satisfaction and outcomes of current state of care transitions in which we have involved patients, significant others, and healthcare professionals in several care trajectories[20, 21].

Patient and public involvement

This study protocol for a feasibility study includes a description of the development of the intervention. The intervention was developed using a co-design methodology that involved five workshops followed by continuous discussions with people with stroke, a significant

For peer review only - http://bmjopen.bmj.com/site/about/guidelines.xhtml

other, and health care professionals from hospital and primary care rehabilitation. The co-design included joint development of the components of the intervention, contextual factors to consider, needs of participants, and important outcomes to target.

Study design

Feasibility study, with a non-randomized controlled study design. The intervention will be implemented at a geriatric stroke unit and an acute stroke unit in hospital A in Stockholm, Sweden. The controls will be recruited from a geriatric stroke unit and an acute stroke unit at hospital B in Stockholm, Sweden. Thereto, we have historic controls with pre-data from the same units from our previous studies at hospital A and B. This design will allow us to compare intervention patients with matched controls, the intervention patients with matched historic controls, and the control patients with historic controls. Controls will be matched regarding age, gender, stroke severity and civil status (living alone or co-habiting).

Participants

Patients and significant others

Inclusion criteria: Patients who have had a first time ever or recurrent stroke and who are to be discharged from the participating stroke units to home and referred to a rehabilitation team in primary healthcare for continued rehabilitation in the home. The patients will be informed of the study and invited to participate at the hospital by a research assistant. The research assistant will provide oral and written information about the study and obtain consent to participate.

For peer review only - http://bmjopen.bmj.com/site/about/guidelines.xhtml

Sample size

No power calculation was conducted for this feasibility study, as the primary aim is to assess feasibility of the study. The likely effectiveness will be used for power calculation for a future large-scale trial. In total 50 persons will be consecutively included, 25 patients from the intervention site and 25 patients from the control site will be recruited. Included patients will be asked to name a significant other to be invited to also participate in the study. Patients who do not have or do not want to name a significant other will remain included.

Healthcare professionals

A purposive sampling of healthcare professionals (in terms of hospital employees or home rehabilitation employees, profession, years in profession) who have participated in the delivery of the intervention will be invited to participate.

Care transitions in control group

Our previous studies identified that a rehabilitation transition "as usual" is initiated by an electronic referral from the hospital healthcare professionals to the receiving neurorehabilitation team; the referral notifies the neurorehabilitation team about the patient and of the discharge; and the neurorehabilitation team is obliged to initiate contact with the patient within 48 hours from referral and/or hospital discharge.

Data collection

RQ1 and RQ2

Data on acceptability and fidelity of the intervention will be collected by participant observations, interviews, and data from the healthcare record. In the intervention site, observations will be conducted of the "what matters to me"-dialogue, the bridging e-meeting, and discharge encounters. The observations will be conducted using a structured protocol in

which the following criteria will be assessed: use of plain language, open-ended questions versus yes/no questions, asking patients to repeat information in own words, repeat/revise information when needed. Individual interviews with the participating healthcare professionals and patients will be conducted after these observations. Healthcare professionals interviews will target perspectives on patient understanding of information; own understanding of patient needs, resources and will; experiences of collaboration with patients and healthcare professionals; and experience of the “what matters to me”-dialogue, the bridging e-meeting, and discharge encounters. Patient interviews will target perspectives on own understanding of information material; how healthcare professionals explained information; experiences of collaboration with healthcare professionals; and how own perspectives and resources were considered.

After all 25 patients have been discharged with the co-designed rehabilitation link, healthcare professionals at the intervention site will be interviewed in focus groups on their overall understanding of the intervention including the training, the experience of delivering the intervention, and their perspectives on content of the intervention. Patients and significant others at both intervention and control sites will be individually interviewed on their experiences of communication with healthcare professionals at hospital, how their needs were taken into consideration and, and their satisfaction with and preparedness for the discharge and the home coming. All interviews will be audio-recorded and transcribed verbatim.

Data on number of e-meetings and instant messages, and content of the coordinated rehabilitation plan will be collected from the healthcare records of the included patients.

RQ3

Data on likely effectiveness will be collected using questionnaires and registry data.

Baseline data on participants' characteristics, length of hospital stay, disease-related data, and functioning in everyday life will be retrieved from medical records. Data on our primary outcome satisfaction with co-designed rehabilitation link will be collected with the Care Transition Measure [28, 29]. Data on our secondary outcome health literacy will be collected with the Health Literacy Questionnaire[30]. These questionnaires together with an envelope (addressed and postage paid) will be sent by mail within one week after discharge from the hospital.

Data on patients' and significant others' outcomes at three months after stroke will be collected using questionnaires in structured interviews. Data will be collected from people with stroke on: health literacy[30], medication adherence[31], cognitive function[32], signs of anxiety and depression[33], fatigue[34], perceived impact of stroke on function, activities and participation in everyday life[35], need of assistance in performing personal activities of daily living[36], participation in complex and social activities[37], health-related quality of life[38], and self-efficacy[39]. Data on use of healthcare will be obtained from the register of healthcare contacts at Region Stockholm. Data on caregiver burden[40], and informal care supplied will be collected using questionnaires in structured interviews with significant others. The same reliable and validated measures and points in time for data collection have been applied in the pre-study of patient and significant other outcomes in current care transitions.

Analyses

For peer review only - http://bmjopen.bmj.com/site/about/guidelines.xhtml

Observations and interviews targeting acceptability and fidelity of the intervention (i.e. RQ 1-2) will be analyzed using manifest qualitative content analysis[41]. Data on likely effectiveness (RQ3) of the co-designed rehabilitation link will be analyzed with test statistics and compared with data collected from control patients and the historic controls from the pre-study of outcomes and resource use in current transitions. Analysers will be blinded to participant allocation to intervention or control.

Ethics and dissemination

Ethical approvals have been obtained from the Swedish Ethical Review Authority, no. 2015/1923-31/2 and no. 2019-04219. The proposed project will be conducted in accordance to the Declaration of Helsinki; oral and written consent to participate are to be obtained from all participants in the upcoming data collections. All study related information about the participants will be stored securely at the Karolinska Institutet. Only the researcher within the group will have access to data.

The results will be published open-access in peer-reviewed journals. Dissemination also includes presentation at national and international conferences.

Discussion

The primary aim is to assess the feasibility operationalized as fidelity, acceptability, and likely effectiveness of the co-designed rehabilitation link for persons with stroke. The co-designed rehabilitation link will address a poorly functioning part of the care trajectories in current healthcare identified in on our pre-studies[20, 21] and literature on the stroke care trajectory[4, 18]. A recent Cochrane review identified that studies on the discharge planning mainly focused on individualized discharge plans in order to reduce readmissions[17]. Such plans may render a small reduction of hospital readmissions, but the development of care

For peer review only - http://bmjopen.bmj.com/site/about/guidelines.xhtml

transitions have so far not involved the patients or significant others, nor all professionals between whom coordination is required. The development of the co-designed rehabilitation link has therefore involved all these users along the transition process[42]. Such involvement in research has the potential to better identify and re-conceptualize problems and incorporate the experiences of the users[42].

List of abbreviations

ESD - Early Supported Discharge

RQ – research question

DECLERATION

Consent to publish

Not applicable.

Availability of data and materials

Not applicable yet. This study protocol does not contain any data or materials that can be shared. Contact corresponding author for up-dated information on availability of data and materials.

Competing interests

The authors declare that they have no competing interests

Funding

This work was supported by the Kamprad Family Foundation grant number: 20190185, the Doctoral School in Healthcare Sciences grant number: 2-134/2016, the Swedish Stroke

For peer review only

Association grant number: N/A, and Neuro Sweden grant number: N/A. The funders had no and will not have any role in design, data collection, data analysis, interpretation of data or writing of manuscripts.

Author Contributions

MF, SL, MT, ACL, ME, LvK and CY drafted the manuscript. BCB, CW and JH made significant contributions in revising and re-writing the manuscript. All authors have read and approved the manuscript.

Acknowledgements

We would like to thank all participating people with stroke, significant others, and health care professionals for the collaboration in the development of this intervention.

References

1. *Global, regional, and national burden of stroke, 1990-2016: a systematic analysis for the Global Burden of Disease Study 2016.* Lancet Neurol, 2019. **18**(5): p. 439-458.
2. Wissel J, Olver J, Sunnerhagen KS. *Navigating the poststroke continuum of care.* J Stroke Cerebrovasc Dis, 2013. **22**(1): p. 1-8.
3. Tistad M, Ytterberg C, Sjostrand C, et al. *Shorter length of stay in the stroke unit: comparison between the 1990s and 2000s.* Top Stroke Rehabil, 2012. **19**(2): p. 172-81.
4. Langhorne P, Ramachandra S. *Organised inpatient (stroke unit) care for stroke: network meta-analysis.* Cochrane Database Syst Rev, 2020. **4**: p. Cd000197.
5. Aarnes R, Stubberud J, Lerdal A. *A literature review of factors associated with fatigue after stroke and a proposal for a framework for clinical utility.* Neuropsychol Rehabil, 2019: p. 1-28.
6. Connolly T, Mahoney E. *Stroke survivors' experiences transitioning from hospital to home.* J Clin Nurs, 2018. **27**(21-22): p. 3979-3987.
7. Wottrich AW, Astrom K, Lofgren M. *On parallel tracks: newly home from hospital--people with stroke describe their expectations.* Disabil Rehabil, 2012. **34**(14): p. 1218-24.

For peer review only - http://bmjopen.bmj.com/site/about/guidelines.xhtml

8. Almborg AH, Ulander K, Thulin A, et al. *Patients' perceptions of their participation in discharge planning after acute stroke.* Journal of Clinical Nursing, 2009. **18**(2): p. 199-209.
9. Almborg AH, Ulander K, Thulin A, et al. *Discharge planning of stroke patients: the relatives' perceptions of participation*, in *J Clin Nurs*. 2009: England. p. 857-65.
10. Ytterberg C, von Koch L, Erikson A. *Abandoned to the strains of daily life: a qualitative study of the long-term experiences in partners to persons after a mild to moderate stroke.* Disabil Rehabil, 2019. **41**(6): p. 649-655.
11. The Swedish Agency for Health and Care Services Analysis [Vårdanalys]. The Patient Law in practice - a baseline measure [Patientlagen i praktiken – en baslinjemätning]. 2015, Report No.: 2015:1.12.
12. Flink M, Ekstedt M. *Planning for the Discharge, not for Patient Self-Management at Home - An Observational and Interview Study of Hospital Discharge.* Int J Integr Care, 2017. **17**(6): p. 1.
13. Gustafsson L, Bootle K. *Client and carer experience of transition home from inpatient stroke rehabilitation.* Disabil Rehabil, 2013. **35**(16): p. 1380-6.
14. Braet A, Weltens C, Sermeus W. *Effectiveness of discharge interventions from hospital to home on hospital readmissions: a systematic review.* JBI Database System Rev Implement Rep, 2016. **14**(2): p. 106-73.
15. Leppin AL, Gionfriddo MR, Kessler M, et al. *Preventing 30-Day Hospital Readmissions: A Systematic Review and Meta-analysis of Randomized Trials.* JAMA Intern Med, 2014.
16. Hesselink G, Schoonhoven L, Barach P, et al. *Improving patient handovers from hospital to primary care: a systematic review.* Ann Intern Med, 2012. **157**(6): p. 417-28.
17. Goncalves-Bradley DC, Lannin NA, Clemson LM, et al. *Discharge planning from hospital.* Cochrane Database Syst Rev, 2016(1): p. Cd000313.
18. Langhorne P, Baylan S. *Early supported discharge services for people with acute stroke.* Cochrane Database Syst Rev, 2017. **7**: p. Cd000443.
19. Fisher RJ, Gaynor C, Kerr M, et al. *A consensus on stroke: early supported discharge.* Stroke, 2011. **42**(5): p. 1392-7.
20. Lindblom S, Flink M, Sjöstrand S, et al. *Perceived Quality of Care Transitions between Hospital and the Home in People with Stroke.* J Am Med Dir Assoc, 2020.
21. Lindblom S, Ytterberg C, Elf M, et al. *Perceptive Dialogue for Linking Stakeholders and Units During Care Transitions - A Qualitative Study of People with Stroke, Significant Others and Healthcare Professionals in Sweden.* Int J Integr Care, 2020. **20**(1): p. 11.
22. Boyd H, McKernon S, Mullin B, et al. *Improving healthcare through the use of co-design.* N Z Med J, 2012. **125**(1357): p. 76-87.
23. Ha Dinh TT, Bonner A, Clark R, et al. *The effectiveness of the teach-back method on adherence and self-management in health education for people with chronic disease: a systematic review.* JBI Database System Rev Implement Rep, 2016. **14**(1): p. 210-47.
24. Oh EG, Lee HJ, Yang YL, et al. *Effectiveness of Discharge Education With the Teach-Back Method on 30-Day Readmission: A Systematic Review.* J Patient Saf, 2019.
25. Institute of Medicine. *Crossing the quality chasm: a new health system for the twenty-first century*. 2001, National Academies Press: Washington.

For peer review only

26. Valentijn PP. *Rainbow of Chaos: A study into the Theory and Practice of Integrated Primary Care: Pim P. Valentijn, [S.l.: s.n.], 2015 (Print Service Ede), pp. 195, Doctoral Thesis Tilburg University, The Netherlands, ISBN: 978-94-91602-40-5.* Int J Integr Care, 2016. **16**(2): p. 3.
27. Craig P, Dieppe P, Macintyre S, et al. *Developing and evaluating complex interventions: the new Medical Research Council guidance.* Bmj, 2008. **337**: p. a1655.
28. Coleman EA, Smith JD, Frank JC, et al. *Development and testing of a measure designed to assess the quality of care transitions.* Int J Integr Care, 2002. **2**: p. e02.
29. Flink M, Tessma M, Cvancarova Smastuen M, et al. *Measuring care transitions in Sweden: validation of the care transitions measure.* Int J Qual Health Care, 2018. **30**(4): p. 291-297.
30. Osborne RH, Batterham RW, Elsworth GR, et al. *The grounded psychometric development and initial validation of the Health Literacy Questionnaire (HLQ).* BMC Public Health, 2013. **13**: p. 658.
31. Chan AHY, Horne R, Hankins M, et al. *The Medication Adherence Report Scale: A measurement tool for eliciting patients' reports of nonadherence.* Br J Clin Pharmacol, 2019.
32. Nasreddine ZS, Phillips NA, Bedirian V, et al. *The Montreal Cognitive Assessment, MoCA: a brief screening tool for mild cognitive impairment.* J Am Geriatr Soc, 2005. **53**(4): p. 695-9.
33. Zigmond AS, Snaith RP. *The hospital anxiety and depression scale.* Acta Psychiatr Scand, 1983. **67**(6): p. 361-70.
34. Krupp LB, LaRocca NG, Muir-Nash J, et al. *The fatigue severity scale. Application to patients with multiple sclerosis and systemic lupus erythematosus.* Arch Neurol, 1989. **46**(10): p. 1121-3.
35. Duncan PW, Bode RK, Min Lai S, et al. *Rasch analysis of a new stroke-specific outcome scale: the Stroke Impact Scale.* Arch Phys Med Rehabil, 2003. **84**(7): p. 950-63.
36. Mahoney FI, Barthel DW. *FUNCTIONAL EVALUATION: THE BARTHEL INDEX.* Md State Med J, 1965. **14**: p. 61-5.
37. Wade DT, Legh-Smith J, Langton Hewer R. *Social activities after stroke: measurement and natural history using the Frenchay Activities Index.* Int Rehabil Med, 1985. **7**(4): p. 176-81.
38. *EuroQol--a new facility for the measurement of health-related quality of life.* Health Policy, 1990. **16**(3): p. 199-208.
39. Carlstedt E, Lexell EM, Pessah-Rasmussen H, et al. *Psychometric properties of the Swedish version of the General Self-Efficacy Scale in stroke survivors.* Int J Rehabil Res, 2015. **38**(4): p. 333-7.
40. Elmstahl S, Malmberg B, Annerstedt L. *Caregiver's burden of patients 3 years after stroke assessed by a novel caregiver burden scale.* Arch Phys Med Rehabil, 1996. **77**(2): p. 177-82.
41. Graneheim UH, Lundman B. *Qualitative content analysis in nursing research: concepts, procedures and measures to achieve trustworthiness.* Nurse Educ Today, 2004. **24**(2): p. 105-12.
42. Bate P, Robert G. *Experience-based design: from redesigning the system around the patient to co-designing services with the patient.* Qual Saf Health Care, 2006. **15**(5): p. 307-10.

For peer review only

Legends

Figure 1, The co-designed rehabilitation link

For peer review only

For peer review only - http://bmjopen.bmj.com/site/about/guidelines.xhtml

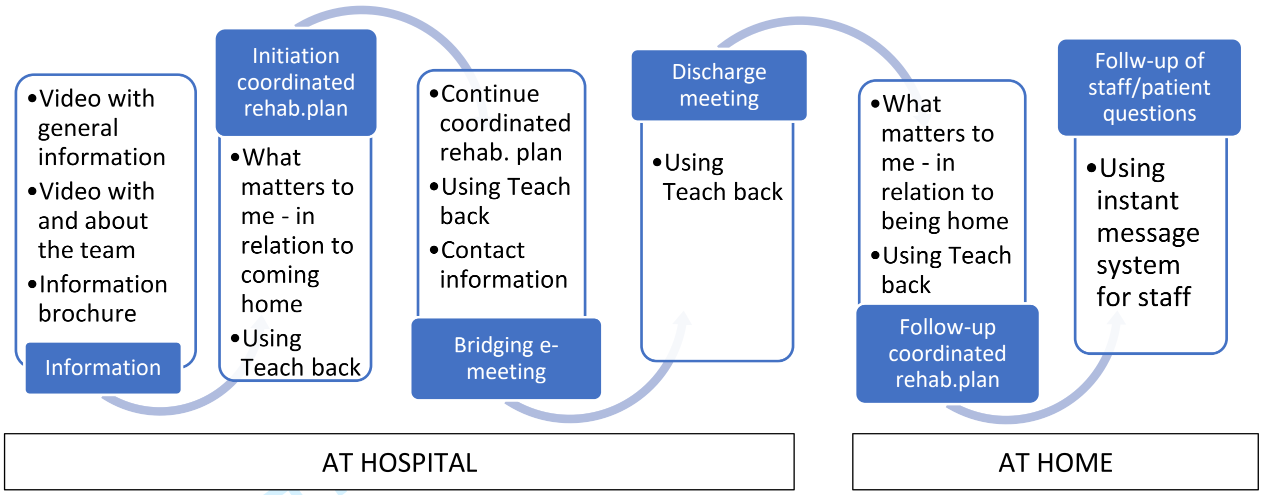

For peer review only

SPIRIT

STANDARD PROTOCOL ITEMS: RECOMMENDATIONS FOR INTERVENTIONAL TRIALS

SPIRIT 2013 Checklist: Recommended items to address in a clinical trial protocol and related documents*

Section/item	ItemNo	Description	
**Administrative information**			
Title	1	Descriptive title identifying the study design, population, interventions, and, if applicable, trial acronym	Title, page 1
Trial registration	2a	Trial identifier and registry name. If not yet registered, name of intended registry	Abstract, Page 2
	2b	All items from the World Health Organization Trial Registration Data Set	N/A
Protocol version	3	Date and version identifier	Abstract, Page 2
Funding	4	Sources and types of financial, material, and other support	Funding, Page 15
Roles and responsibilities	5a	Names, affiliations, and roles of protocol contributors	Title page, Page 1<br>Authors' contributions, page 15
	5b	Name and contact information for the trial sponsor	N/A
	5c	Role of study sponsor and funders, if any, in study design; collection, management, analysis, and interpretation of data; writing of the report; and the decision to submit the report for publication, including whether they will have ultimate authority over any of these activities	Funding, Page 15

For peer review only

	5d	Composition, oles, and responsibilities of the coordinating centre, steering committee, endpoint adjudication committee, data management team, and other individuals or groups overseeing the trial, if applicable (see Item 21a for data monitoring committee)	N/A
**Introduction**			
Background and rationale	6a	Description of research question and justification for undertaking the trial, including summary of relevant studies (published and unpublished) examining benefits and harms for each intervention	Background, Pages 3-5, Aims and hypothesis, page 9
	6b	Explanation for choice of comparators	Study design, Page 10
Objectives	7	Specific objectives or hypotheses	Aims and hypothesis, page 9
Trial design	8	Description of trial design including type of trial (eg, parallel group, crossover, factorial, single group), allocation ratio, and framework (eg, superiority, equivalence, noninferiority, exploratory)	Study design, Page 10
**Methods: Participants, interventions, and outcomes**			
Study setting	9	Description of study settings (eg, community clinic, academic hospital) and list of countries where data will be collected. Reference to where list of study sites can be obtained	Study design, Page 10
Eligibility criteria	10	Inclusion and exclusion criteria for participants. If applicable, eligibility criteria for study centres and individuals who will perform the interventions (eg, surgeons, psychotherapists)	Participants, Pages 10-11

For peer review only

Interventions	11a	Interventions for each group with sufficient detail to allow replication, including how and when they will be administered	Intervention Pages 6-8, Care transitions in control group, Page 11
	11b	Criteria for discontinuing or modifying allocated interventions for a given trial participant (eg, drug dose change in response to harms, participant request, or improving/worsening disease)	N/A
	11c	Strategies to improve adherence to intervention protocols, and any procedures for monitoring adherence (eg, drug tablet return, laboratory tests)	RQ1 and RQ2, Pages 11-12
	11d	Relevant concomitant care and interventions that are permitted or prohibited during the trial	N/A
Outcomes	12	Primary, secondary, and other outcomes, including the specific measurement variable (eg, systolic blood pressure), analysis metric (eg, change from baseline, final value, time to event), method of aggregation (eg, median, proportion), and time point for each outcome. Explanation of the clinical relevance of chosen efficacy and harm outcomes is strongly recommended	RQ3, Pages 12-13
Participant timeline	13	Time schedule of enrolment, interventions (including any run-ins and washouts), assessments, and visits for participants. A schematic diagram is highly recommended (see Figure)	Figure, Pages 6<br>RQ3, Page 13
Sample size	14	Estimated number of participants needed to achieve study objectives and how it was determined, including clinical and statistical assumptions supporting any sample size calculations	Patients and significant others, Page 10
Recruitment	15	Strategies for achieving adequate participant enrolment to reach target sample size	N/A
**Methods: Assignment of interventions (for controlled trials)**			
Allocation:			

Sequence generation	16a	Method of generating the allocation sequence (eg, computer-generated random numbers), and list of any factors for stratification. To reduce predictability of a random sequence, details of any planned restriction (eg, blocking) should be provided in a separate document that is unavailable to those who enrol participants or assign interventions	N/A
Allocation concealment mechanism	16b	Mechanism of implementing the allocation sequence (eg, central telephone; sequentially numbered, opaque, sealed envelopes), describing any steps to conceal the sequence until interventions are assigned	N/A
Implementation	16c	Who will generate the allocation sequence, who will enrol participants, and who will assign participants to interventions	N/A
Blinding (masking)	17a	Who will be blinded after assignment to interventions (eg, trial participants, care providers, outcome assessors, data analysts), and how	N/A
	17b	If blinded, circumstances under which unblinding is permissible, and procedure for revealing a participant's allocated intervention during the trial	N/A
**Methods: Data collection, management, and analysis**			
Data collection methods	18a	Plans for assessment and collection of outcome, baseline, and other trial data, including any related processes to promote data quality (eg, duplicate measurements, training of assessors) and a description of study instruments (eg, questionnaires, laboratory tests) along with their reliability and validity, if known. Reference to where data collection forms can be found, if not in the protocol	Data collection, Pages 11-13
	18b	Plans to promote participant retention and complete follow-up, including list of any outcome data to be collected for participants who discontinue or deviate from intervention protocols	N/A

For peer review only - http://bmjopen.bmj.com/site/about/guidelines.xhtml

Data management	19	Plans for data entry, coding, security, and storage, including any related processes to promote data quality (eg, double data entry; range checks for data values). Reference to where details of data management procedures can be found, if not in the protocol	N/A
Statistical methods	20a	Statistical methods for analysing primary and secondary outcomes. Reference to where other details of the statistical analysis plan can be found, if not in the protocol	Analyses, Page 13
	20b	Methods for any additional analyses (eg, subgroup and adjusted analyses)	N/A
	20c	Definition of analysis population relating to protocol non-adherence (eg, as randomised analysis), and any statistical methods to handle missing data (eg, multiple imputation)	N/A
**Methods: Monitoring**			
Data monitoring	21a	Composition of data monitoring committee (DMC); summary of its role and reporting structure; statement of whether it is independent from the sponsor and competing interests; and reference to where further details about its charter can be found, if not in the protocol. Alternatively, an explanation of why a DMC is not needed	N/A
	21b	Description of any interim analyses and stopping guidelines, including who will have access to these interim results and make the final decision to terminate the trial	N/A
Harms	22	Plans for collecting, assessing, reporting, and managing solicited and spontaneously reported adverse events and other unintended effects of trial interventions or trial conduct	N/A
Auditing	23	Frequency and procedures for auditing trial conduct, if any, and whether the process will be independent from investigators and the sponsor	N/A
**Ethics and dissemination**			

Research ethics approval	24	Plans for seeking research ethics committee/institutional review board (REC/IRB) approval	Ethics approval and consent to participate, Page 14-15
Protocol amendments	25	Plans for communicating important protocol modifications (eg, changes to eligibility criteria, outcomes, analyses) to relevant parties (eg, investigators, REC/IRBs, trial participants, trial registries, journals, regulators)	Methods, Page 9
Consent or assent	26a	Who will obtain informed consent or assent from potential trial participants or authorised surrogates, and how (see Item 32)	Patient and significant other, Page 10
	26b	Additional consent provisions for collection and use of participant data and biological specimens in ancillary studies, if applicable	N/A
Confidentiality	27	How personal information about potential and enrolled participants will be collected, shared, and maintained in order to protect confidentiality before, during, and after the trial	Ethics approval and consent to participate, Page 14-15
Declaration of interests	28	Financial and other competing interests for principal investigators for the overall trial and each study site	Funding, Page 15
Access to data	29	Statement of who will have access to the final trial dataset, and disclosure of contractual agreements that limit such access for investigators	Ethics approval and consent to participate, Page 14-15
Ancillary and post-trial care	30	Provisions, if any, for ancillary and post-trial care, and for compensation to those who suffer harm from trial participation	N/A
Dissemination policy	31a	Plans for investigators and sponsor to communicate trial results to participants, healthcare professionals, the public, and other relevant groups (eg, via publication, reporting in results databases, or other data sharing arrangements), including any publication restrictions	N/A
	31b	Authorship eligibility guidelines and any intended use of professional writers	N/A

For peer review only - http://bmjopen.bmj.com/site/about/guidelines.xhtml

	31c	Plans, if any, for granting public access to the full protocol, participant-level dataset, and statistical code	N/A
**Appendices**			
Informed consent materials	32	Model consent form and other related documentation given to participants and authorised surrogates	N/A
Biological specimens	33	Plans for collection, laboratory evaluation, and storage of biological specimens for genetic or molecular analysis in the current trial and for future use in ancillary studies, if applicable	N/A

*It is strongly recommended that this checklist be read in conjunction with the SPIRIT 2013 Explanation & Elaboration for important clarification on the items. Amendments to the protocol should be tracked and dated. The SPIRIT checklist is copyrighted by the SPIRIT Group under the Creative Commons "Attribution-NonCommercial-NoDerivs 3.0 Unported" license.

For peer review only

BMJ Open

Person-centred care transitions for people with stroke: study protocol for evaluation of feasibility of a co-designed care transition support

Journal:	*BMJ Open*
Manuscript ID	bmjopen-2020-047329.R1
Article Type:	Protocol
Date Submitted by the Author:	20-May-2021

Complete List of Authors:	Flink, Maria; Karolinska Institutet, Neurobiology, Care Sciences and Society; Karolinska University Hospital<br>Lindblom, Sebastian; Karolinska Institutet, Neurobiology, Care Sciences and Society; Karolinska University Hospital<br>Tistad, Malin; Dalarna University<br>Laska, Ann Charlotte ; Karolinska Institutet, Department of Clinical Sciences Danderyd Hospital<br>Bertilsson, Bo Christer; Karolinska Institutet, Neurobiology, Care Sciences and Society<br>Wärlinge, Carmen; Region Stockholm, Academic Primary Health Care Centre<br>Hasselström, Jan; Karolinska Institutet, 6. Department of Neurobiology, Care Sciences and Society; Region Stockholm, Academic Primary Health Care Centre<br>Elf, Marie; Dalarna University, School of education, health and social studies<br>von Koch, Lena; Karolinska Institutet, Neurobiology, Care Sciences and Society; Karolinska University Hospital<br>Ytterberg, Charlotte; Karolinska Institutet, Neurobiology, Care Sciences and Society; Karolinska University Hospital

**Primary Subject Heading**:	Neurology
Secondary Subject Heading:	Patient-centred medicine, Rehabilitation medicine
Keywords:	Stroke < NEUROLOGY, REHABILITATION MEDICINE, Organisation of health services < HEALTH SERVICES ADMINISTRATION & MANAGEMENT

SCHOLARONE™
Manuscripts

Person-centred care transitions for people with stroke: study protocol for evaluation of feasibility of a co-designed care transition support

Maria Flink[1,2], Sebastian Lindblom[1,2], Malin Tistad[1,3], Ann Charlotte Laska[4], Bo Christer Bertilson[1], Carmen Wärlinge[5], Jan Hasselström[1,5], Marie Elf[3], Lena von Koch[1,2], Charlotte Ytterberg[1,2]

1. Karolinska Institutet, Department of Neurobiology, Care Sciences and Society, Huddinge, Sweden
2. Karolinska University Hospital, Stockholm, Sweden
3. Dalarna University, School of Education, Health and Social Studies, Falun, Sweden
4. Karolinska Institutet, Department of Clinical Sciences Danderyd Hospital, Stockholm, Sweden
5. Academic Primary Health Care Centre, Stockholm Health Care Services (SLSO), Region Stockholm, Sweden

Corresponding author:

Maria Flink

Alfred Nobels allé 23, D2

Karolinska Institutet

SE- 14183 Huddinge, SWEDEN

maria.flink@ki.se

Abstract

Background: Care transitions after stroke should be bridged through collaboration between hospital staff and home rehabilitation teams, and that well-coordinated transitions can reduce death and disability after a stroke. Despite this, health services are delivered within

For peer review only

organizational structures rather than based on patients' needs. The aim is to assess the feasibility operationalized as fidelity and acceptability of a co-designed care transition support for people with stroke.

Methods: This study protocol describes the evaluation of a feasibility study using a non-randomized controlled design. The co-designed care transition support includes patient information using videos, leaflets and teach back; what-matters-to me dialogue; coordinated rehabilitation plan; bridging e-meeting; and a message system for cross-organizational collaboration. The care transition support will be implemented at a geriatric stroke unit and an acute stroke unit. Patients with stroke, first time ever or recurrent, who are to be discharged from hospital to home and referred to a rehabilitation team in primary healthcare for continued rehabilitation in the home will be included. One week after stroke, data will be collected on the primary outcome satisfaction with the care transition support, and on the secondary outcome health literacy and medication adherence. After three months, patients' and significant others' outcomes will be collected through structured face-to-face interviews; data on use of healthcare will be obtained from a register of healthcare contacts. Patient and significant other outcomes will be compared with matched controls from another geriatric stroke unit and acute stroke unit, and with matched historic controls from a previous dataset at the intervention and control units. Data on acceptability and fidelity will be assessed through interviews and observations at the intervention units.

Discussion: The co-designed care transition support addresses a poorly functioning part of the care trajectories in current healthcare

Trial registration: http://www.clinicaltrials.gov id: NCT02925871. Date of registration October 6, 2016

Protocol version: 1

Strengths and limitations of this study

- The intervention was developed following the guidelines of the Medical Research Council
- The intervention was developed using co-design methodology with people with stroke, significant other, and health care professionals
- The use of mixed methods will provide knowledge of both process and outcomes
- The study is non-randomised, but the intervention group will be compared to matched controls

Key words

Stroke, care planning, continuity of patient care, implementation, participation, patient satisfaction, rehabilitation, self-efficacy, significant others, transitional care, user involvement

Background

Stroke, one of the leading causes of disability(1), has an abrupt onset and is a stressful event, both for the person with stroke, and the family(2). The past decades have seen improvements in medical and acute care of stroke as well as a reduction of the mean length of hospital stay in stroke units in high income countries(3). Strong evidence and consensus have shown that stroke care should initially be supplied in stroke units in hospital in the acute phase(4), followed by a period of rehabilitation to regain functioning and to receive psychosocial support(2). Hence, a care transition – a shift in responsibility from one healthcare setting to another –is almost always needed. This may entail a care transition to inpatient rehabilitation

peer review only

or directly to rehabilitation in the home provided by primary healthcare, e.g., by a multi-professional team.

Uncoordinated care transitions impose a burden to patients and their significant others, especially when they lack information to navigate the healthcare system(2). Cognitive impairments, post-stroke fatigue and depression(5), aphasia as well as the sudden onset, often render the person having had the stroke and their significant others unprepared for the care transition to home(6, 7). In addition, a short hospital stay, little time to participate in transition planning(8, 9), and a fragmented healthcare may contribute to patients having unrealistic expectations(7) and a sense of being abandoned(10) in a new and complex life situation. Although Swedish laws and other regulations require care providers to coordinate care, a great responsibility is put on the patient and significant others to coordinate their own care(11), while few arrangements are offered to support a patient's ability to participate in their own care or to self-manage(12, 13).

Several care transition interventions have been developed, both for people with stroke and for other populations. Strong evidence supports that to reduce re-hospitalizations, such care transitions must include both pre- and post-discharge activities and support patients' capacity for self-management(14, 15). Such pre- and post-discharge activities include discharge planning, structured discharge information, coordinated follow-up, and timely communication between care providers(16, 17). For people with stroke, extensive evidence shows that the rehabilitation care transition Early Supported Discharge (ESD) can reduce death and disability after a mild to moderate stroke(18). ESD implies that a stroke specific, multidisciplinary team plans and coordinates the hospital discharge, and provides rehabilitation in the community(19). Despite the evidence, ESD has scarcely been

For peer review only - http://bmjopen.bmj.com/site/about/guidelines.xhtml

implemented in Sweden. A possible explanation for the lack of implementation is that cross-organizational health services such as ESD may be difficult to accomplish in fragmented healthcare systems with firm borders between hospital and community care.

There is hence a need for a rehabilitation care transition that both takes the patient's needs and capacity, and the organizational settings into consideration. This need was highlighted in our prospective observational study of 206 people with acute stroke, of the current state of transitions from stroke and geriatric hospital units to the home with subsequent rehabilitation in primary healthcare in Stockholm, Sweden(20, 21) conducted from April 2016 to February 2018. One week after hospital discharge the participants were followed-up regarding satisfaction with care transition and at three- and 12-months regarding functioning in everyday life and use of healthcare. In addition, we performed a grounded theory study of perceptions of current care transitions of patients, families, and healthcare professionals. The findings from the pre-studies(20, 21) identified a need for improved dialogue between patients and professionals; between professionals working in the same care provider organization; and between professionals in different organizations. The improved dialogue includes a communication where all parties are involved on equal terms and strive for a common understanding. Further, a need to enhance patients' preparedness for discharge, and for their self-management at home was identified. Based on the results(20, 21) we identified a need to develop bridging communicative links along the transition process, within and between care provider organizations and between patients and healthcare professionals. This aims to ensure a coordinated rehabilitation transition, and patients and significant others to feel prepared for the home coming.

For peer review only

Development of the intervention

The intervention is hence based both on our pre-studies(20, 21) and on literature on the stroke care trajectory(4, 18), and was developed in a co-design process in two steps(22).

First, five co-design workshops led by a trained facilitator were held with people with stroke, a significant other, and healthcare professionals from hospital stroke units and multidisciplinary home rehabilitation teams. Three areas of unmet needs related to care transitions were identified across patients, significant others, and healthcare professionals:
1) Shared understanding of patient illness and situation: repeated information about the present situation and expected future; communication adapted to the situation; updated written information; time to ask questions; structured and timely information.
2) Preparedness for home-coming: feeling safe and understood in the discharge; clarity about services provided by the home rehabilitation team; being prepared for the home-coming; clarity about medications; own perspective and needs taken into consideration.
3) Coordination: coordination between stroke unit and home rehabilitation teams; clarity about the care trajectory; collaboration between sites.
Based on these needs, suggestions for improvements were co-designed by the participants.

Thereafter, interactive e-meetings with healthcare professionals were held in which suggestions from the co-design workshops were further developed and contextually adapted into the intervention.

For peer review only

The intervention – the co-designed care transition support

The intervention consists of several elements that aim to meet patients', significant others', and professionals' need of shared understanding, patient preparedness for home-coming, and coordination (Figure 1).

Please insert Figure about here.

Three different sources of information of home-rehabilitation will be offered to patients and significant others: *two information videos and one leaflet*. First, an information video with general information of home-rehabilitation will be shown during daytime in the day room. Second, an information video with the professionals in the patient's specific home-rehabilitation team will be provided to patients on tablets. Third, an information leaflet with general information about home-rehabilitation, a link to the information video, and contact information to the specific team. After the patient has accessed these sources of information the hospital staff will initiate a dialogue with the patient of '*what matters to me'* in relation to coming home with continued rehabilitation. In this dialogue the hospital staff and the patient will initate the development of a *coordinated rehabilitation plan*, based on the patient's needs and wishes for the continuing rehabilitation at home, as well as on the hospital staffs' assessment. Based on agreements in the initiated plan, the hospital staff will write a referral to the home-rehabilitation team and book an e-meeting with the team before discharge. This *bridging e-meeting* aims to connect the patient with the team that will be responsible for rehabilitation at home through a first personal contact and decide an appointment for the first home-visit. The e-meetings will include a dialogue between patient, the significant other, and the hospital staff and the rehabilitation team. The dialogue will include the patient's situation, needs, wishes, and fears in relation to coming home; a presentation from the home

For peer review only - http://bmjopen.bmj.com/site/about/guidelines.xhtml

rehabilitation team of their services; a possibility for the patient and significant other to ask questions; and to continue the initiated coordinated rehabilitation plan for the homecoming. The e-meeting will function as an in-reaching bridge between patient, hospital staff, and the home-rehabilitation team. Thereto, the hospital staff will add contact information, i.e., the telephone number to the home rehabilitation team in the patient's cell phone, and make sure that the correct contact information to the patient and significant other is available to the home rehabilitation team. After coming home, the home-rehabilitation team, the patient, and significant others will review and revise the initiated coordinated rehabilitation plan.

Teach back(23, 24) is a person-centred, iterative communication method focused on shared understanding of the patient's situation and of the professional's information. The aim of *Teach back* is both to ensure that health professionals have understood the patient correctly and an improved patient learning and understanding of the own health condition, medications, and how to navigate the healthcare system, i.e., improved health literacy. Teach-back will be used in the *'what matters to me'*-dialogue, the bridging e-meeting, and discharge encounters. The professionals will give information and thereafter ask the patient to tell in own words what was said, emphasizing that this is to check how the professional explained. The detailed discharge information entails information on follow-up, what to consider after discharge, and where to turn with questions.

A safe *instant messaging system* within the electronical health record will ensure inter-organizational communication between healthcare professionals. This will ensure exchange of information and resolve remaining questions on the patient's care between the hospital and home-rehabilitation, such as revoked driving license or medications.

For peer review only

The co-designed care transition support is grounded in principals of person-centred care(25) and of integrated care(26) on a micro and meso level. This entails that the development of the intervention and the actual intervention both are based on the individual's preferences, needs, and values, taking patient resources into consideration, i.e. is person-centred(25), and strives to be realistic regarding what can be provided by the healthcare professionals in the involved organizational settings. We adhere to the person-centred Rainbow model of integrated care(26). This model emphasizes the need for both functional and normative links between patients and professionals, as well as within and between hospital and primary care organizations. Functional here refers to the information systems that link the hospital and primary care organizations. Normative here refers to shared understanding and values, both within and between organizations.

Care transitions in control group

Our previous studies identified that a rehabilitation care transition 'as usual' is initiated by an electronic referral from the hospital healthcare professionals to the receiving neurorehabilitation team. The referral notifies the neurorehabilitation team about the patient and of the discharge, The neurorehabilitation team is obliged to initiate contact with the patient within 48 hours from referral and/or hospital discharge.

Aims and hypothesis

The primary aim is to assess the feasibility operationalized as fidelity and acceptability of a co-designed care transition support for people with stroke.

The following research questions (RQ) will be used:

RQ1: Is the intervention delivered as intended (fidelity)?

RQ2: Is the intervention acceptable regarding content and delivery?

RQ3: Are there any differences in patient and significant other outcomes between patients who received the intervention and matched controls, and between intervention patients and matched historic controls?

We hypothesise that information through a person-centred dialogue with focus on shared understanding between patients and healthcare professionals and a discharge focused on bridging the hospitalization with the home through meetings and coordinated rehabilitation plan will help patients and significant others feel more satisfied with the transition and be better prepared for the homecoming including the subsequent rehabilitation activities. This may in turn help patients regain functioning and reduce healthcare utilization.

Methods

This study protocol describes the evaluation of feasibility for the co-designed care transition support using a non-randomized controlled design. Up-dates on the study protocol will be published on Clinical Trials.

The project adheres to the framework for development and evaluation of complex interventions by the Medical Research Council, UK(27), which calls for phased and iterative approaches in the design and evaluation of a complex intervention. This project hence draws upon our pre-studies of the perceptions, satisfaction, and outcomes of current state of care transitions in which we have involved patients, significant others, and healthcare professionals in several care trajectories(20, 21).

For peer review only - http://bmjopen.bmj.com/site/about/guidelines.xhtml

Patient and public involvement

This study protocol for a feasibility study includes a description of the development of the intervention. The intervention was developed using a co-design methodology that involved five workshops followed by continuous discussions with people with stroke, a significant other, and healthcare professionals from hospital and primary care rehabilitation. The co-design included joint development of the components of the intervention, contextual factors to consider, needs of participants, and important outcomes to target.

Study design

Feasibility study, with a non-randomized controlled study design. The intervention will be implemented at a geriatric stroke unit and an acute stroke unit in hospital A in Stockholm, Sweden. The controls will be recruited from a geriatric stroke unit and an acute stroke unit at hospital B in Stockholm, Sweden. Thereto, we have historic controls with pre-data from the same units from our previous studies at hospital A and B (the pre-study conducted from 2016-2018). This design will allow us to compare intervention patients with matched controls, the intervention patients with matched historic controls, and the control patients with historic controls. Controls will be matched according to age, gender, stroke severity and civil status (living alone or co-habiting).

Participants

Patients

Inclusion criteria: Patients who have had a first time ever or recurrent stroke, who are to be discharged from the participating stroke units to home and referred to a rehabilitation team in

For peer review only - http://bmjopen.bmj.com/site/about/guidelines.xhtml

primary healthcare for continued rehabilitation in the home, and who are able to give informed consent by themselves or through a significant other. Most of the patients discharged from these units to home have mild to moderate stroke(28). The patients will be informed of the study and invited to participate at the hospital by a research assistant. The research assistant will provide oral and written information about the study and obtain consent to participate.

Sample size

In total 50 persons will be consecutively included, 25 patients from the intervention site and 25 patients from the control site.

Significant others

Significant others will be included via the included patients. The included patients will be asked to name a significant other to be invited to also participate in the study. The significant others will be mailed written information about the study including an informed consent and a pre-stamped envelope. Significant others who return a signed consent will be included in the study. Patients who do not have or do not want to name a significant other will remain included.

Healthcare professionals

Hospital staff and home rehabilitation team members who have delivered the intervention will be invited to participate in the study. A purposive sampling (in terms of profession, years in profession) will be used. Approximately 15 hospital and home rehabilitation professionals will be included.

Data collection

RQ1 and RQ2

Data on acceptability and fidelity of the intervention will be collected by participant observations, interviews, and data from the healthcare record. In the intervention site, observations will be conducted of the 'what matters to me'-dialogue, the bridging e-meeting, and discharge encounters. The observations will be conducted using a structured protocol in which the following criteria will be assessed: use of plain language, open-ended questions versus yes/no questions, asking patients to repeat information in own words, repeat/revise information when needed. Individual interviews with the participating healthcare professionals and patients will be conducted after these observations. Interviews with healthcare professionals will target perspectives on patient understanding of information; own understanding of patient needs, resources and will; experiences of collaboration with patients and healthcare professionals; and experience of the 'what matters to me'-dialogue, the bridging e-meeting, and discharge encounters. Patient interviews will target perspectives on own understanding of information material; how healthcare professionals explained information; experiences of collaboration with healthcare professionals; and how own perspectives and resources were considered.

After the 25 patients have been discharged with the co-designed care transition support, healthcare professionals at the intervention site will be interviewed in focus groups on their overall understanding of the intervention including the training, the experience of delivering the intervention, and their perspectives on content of the intervention. Patients and significant others at both intervention and control sites will be individually interviewed on their experiences of communication with healthcare professionals at hospital, how their needs were

taken into consideration and, and their satisfaction with and preparedness for the discharge and the home coming. All interviews will be audio-recorded and transcribed verbatim.

Data on number of e-meetings and instant messages, and content of the coordinated rehabilitation plan will be collected from the healthcare records of the included patients.

RQ3

Data on likely effectiveness will be collected using questionnaires and registry data.

Baseline data on participants' characteristics, length of hospital stay, disease-related data, and functioning in everyday life will be retrieved from medical records. Data on our primary outcome satisfaction with co-designed care transition support will be collected with the Care Transition Measure (29, 30). Data on our secondary outcome health literacy will be collected with the Health Literacy Questionnaire(31). These questionnaires together with an envelope (addressed and postage paid) will be sent by mail within one week after discharge from the hospital.

Data on patients' and significant others' outcomes at three months after stroke will be collected using questionnaires in structured interviews. Data will be collected from people with stroke on: health literacy(31), medication adherence(32), cognitive function(33), signs of anxiety and depression(34), fatigue(35), perceived impact of stroke on function, activities and participation in everyday life(36), need of assistance in performing personal activities of daily living(37), participation in complex and social activities(38), health-related quality of life(39), and self-efficacy(40). Data on use of healthcare will be obtained from the register of healthcare contacts at Region Stockholm. Data on caregiver burden(41), and informal care supplied will be collected using questionnaires in structured interviews with significant

For peer review only

others. The same reliable and validated measures and points in time for data collection have been applied in the pre-study of patient and significant other outcomes in current care transitions.

Analyses

Observations and interviews will be analyzed using qualitative content analysis(42). Qualitative content analysis is a method that is well suited for descriptive qualitative analysis(42). Likely effectiveness of the co-designed care transition support (RQ3) in terms of outcomes and resource use, will be examined statistically and compared with control and pre-study historic data. The statistician performing analysis will be blinded to group allocation. Ethics and dissemination

Ethical approvals have been obtained from the Swedish Ethical Review Authority, no. 2015/1923-31/2, and no. 2019-04219. The proposed project will be conducted in accordance with the Declaration of Helsinki; oral and written consent to participate will be obtained from all participants. All study related information about the participants will be stored securely at the Karolinska Institutet. Only the researcher within the group will have access to data. The results will be published open-access in peer-reviewed journals. Dissemination also includes presentation at national and international conferences.

Discussion

The primary aim is to assess the feasibility operationalized as fidelity and acceptability of the co-designed care transition support for people with stroke. The co-designed care transition support will address a poorly functioning part of the care trajectories in current healthcare, as

review only

identified in on our pre-studies(20, 21) and literature on the stroke care trajectory(4, 18). A recent Cochrane review identified that studies on the discharge planning mainly focused on individualized discharge plans in order to reduce readmissions(17). Such plans may render a small reduction of hospital readmissions, but the development of care transitions have so far not involved the patients or significant others, nor all professionals between whom coordination is required. The development of this co-designed care transition support has therefore involved all these users (43). Such involvement has the potential to better identify and re-conceptualize problems and incorporate the experiences of the users(43).

List of abbreviations

ESD - Early Supported Discharge

RQ – research question

Declaration

Consent to publish

Not applicable.

Availability of data and materials

Not applicable yet. This study protocol does not contain any data or materials that can be shared. Contact corresponding author for up-dated information on availability of data and materials.

For peer review only

Competing interests

The authors declare that they have no competing interests

Funding

This work was supported by the Kamprad Family Foundation grant number [20190185], the Doctoral School in Healthcare Sciences [2-134/2016], the Swedish Stroke Association and Neuro Sweden. The funders had no and will not have any role in design, data collection, data analysis, interpretation of data or writing of manuscripts.

Authors' contributions

MF, SL, MT, ACL, ME, LvK and CY drafted the manuscript. BCB, CW and JH made significant contributions in revising and re-writing the manuscript. All authors have read and approved the manuscript.

Acknowledgements

Not applicable

References

1. Global, regional, and national burden of stroke, 1990-2016: a systematic analysis for the Global Burden of Disease Study 2016. Lancet Neurol. 2019;18(5):439-58.
2. Wissel J, Olver J, Sunnerhagen KS. Navigating the poststroke continuum of care. Journal of stroke and cerebrovascular diseases : the official journal of National Stroke Association. 2013;22(1):1-8.
3. Tistad M, Ytterberg C, Sjostrand C, Holmqvist LW, von Koch L. Shorter length of stay in the stroke unit: comparison between the 1990s and 2000s. Topics in stroke rehabilitation. 2012;19(2):172-81.
4. Langhorne P, Ramachandra S. Organised inpatient (stroke unit) care for stroke: network meta-analysis. Cochrane Database Syst Rev. 2020;4:Cd000197.

For peer review only - http://bmjopen.bmj.com/site/about/guidelines.xhtml

5. Aarnes R, Stubberud J, Lerdal A. A literature review of factors associated with fatigue after stroke and a proposal for a framework for clinical utility. Neuropsychological rehabilitation. 2019:1-28.
6. Connolly T, Mahoney E. Stroke survivors' experiences transitioning from hospital to home. J Clin Nurs. 2018;27(21-22):3979-87.
7. Wottrich AW, Astrom K, Lofgren M. On parallel tracks: newly home from hospital--people with stroke describe their expectations. Disability and rehabilitation. 2012;34(14):1218-24.
8. Almborg AH, Ulander K, Thulin A, Berg S. Patients' perceptions of their participation in discharge planning after acute stroke. Journal of Clinical Nursing. 2009;18(2):199-209.
9. Almborg AH, Ulander K, Thulin A, Berg S. Discharge planning of stroke patients: the relatives' perceptions of participation. J Clin Nurs. 18. England2009. p. 857-65.
10. Ytterberg C, von Koch L, Erikson A. Abandoned to the strains of daily life: a qualitative study of the long-term experiences in partners to persons after a mild to moderate stroke. Disability and rehabilitation. 2019;41(6):649-55.
11. The Swedish Agency for Health and Care Services Analysis [Vårdanalys]. The Patient Law in practice - a baseline measure [Patientlagen i praktiken – en baslinjemätning]. 2015, Report No.: 2015:1.
12. Flink M, Ekstedt M. Planning for the Discharge, not for Patient Self-Management at Home - An Observational and Interview Study of Hospital Discharge. Int J Integr Care. 2017;17(6):1.
13. Gustafsson L, Bootle K. Client and carer experience of transition home from inpatient stroke rehabilitation. Disability and rehabilitation. 2013;35(16):1380-6.
14. Braet A, Weltens C, Sermeus W. Effectiveness of discharge interventions from hospital to home on hospital readmissions: a systematic review. JBI Database System Rev Implement Rep. 2016;14(2):106-73.
15. Leppin AL, Gionfriddo MR, Kessler M, Brito JP, Mair FS, Gallacher K, et al. Preventing 30-Day Hospital Readmissions: A Systematic Review and Meta-analysis of Randomized Trials. JAMA internal medicine. 2014.
16. Hesselink G, Schoonhoven L, Barach P, Spijker A, Gademan P, Kalkman C, et al. Improving patient handovers from hospital to primary care: a systematic review. Annals of internal medicine. 2012;157(6):417-28.
17. Goncalves-Bradley DC, Lannin NA, Clemson LM, Cameron ID, Shepperd S. Discharge planning from hospital. Cochrane Database Syst Rev. 2016(1):Cd000313.
18. Langhorne P, Baylan S. Early supported discharge services for people with acute stroke. Cochrane Database Syst Rev. 2017;7:Cd000443.
19. Fisher RJ, Gaynor C, Kerr M, Langhorne P, Anderson C, Bautz-Holter E, et al. A consensus on stroke: early supported discharge. Stroke; a journal of cerebral circulation. 2011;42(5):1392-7.
20. Lindblom S, Flink M, Sjöstrand S, Laska A, von Koch L, Ytterberg C. Perceived Quality of Care Transitions between Hospital and the Home in People with Stroke. J Am Med Dir Assoc. 2020.
21. Lindblom S, Ytterberg C, Elf M, Flink M. Perceptive Dialogue for Linking Stakeholders and Units During Care Transitions - A Qualitative Study of People with Stroke, Significant Others and Healthcare Professionals in Sweden. Int J Integr Care. 2020;20(1):11.
22. Boyd H, McKernon S, Mullin B, Old A. Improving healthcare through the use of co-design. N Z Med J. 2012;125(1357):76-87.

For peer review only - http://bmjopen.bmj.com/site/about/guidelines.xhtml

23. Ha Dinh TT, Bonner A, Clark R, Ramsbotham J, Hines S. The effectiveness of the teach-back method on adherence and self-management in health education for people with chronic disease: a systematic review. JBI Database System Rev Implement Rep. 2016;14(1):210-47.
24. Oh EG, Lee HJ, Yang YL, Kim YM. Effectiveness of Discharge Education With the Teach-Back Method on 30-Day Readmission: A Systematic Review. J Patient Saf. 2019.
25. Institute_of_Medicine. Crossing the quality chasm: a new health system for the twenty-first century. Washington: National Academies Press; 2001.
26. Valentijn PP. Rainbow of Chaos: A study into the Theory and Practice of Integrated Primary Care: Pim P. Valentijn, [S.l.: s.n.], 2015 (Print Service Ede), pp. 195, Doctoral Thesis Tilburg University, The Netherlands, ISBN: 978-94-91602-40-5. Int J Integr Care. 2016;16(2):3.
27. Craig P, Dieppe P, Macintyre S, Michie S, Nazareth I, Petticrew M. Developing and evaluating complex interventions: the new Medical Research Council guidance. Bmj. 2008;337:a1655.
28. Lindblom S. Understanding the links: The exploration of care transitions between hospital and continued rehabilitation in the home after stroke. Stockholm, Sweden: Karolinska Institutet; 2021.
29. Coleman EA, Smith JD, Frank JC, Eilertsen TB, Thiare JN, Kramer AM. Development and testing of a measure designed to assess the quality of care transitions. Int J Integr Care. 2002;2:e02.
30. Flink M, Tessma M, Cvancarova Smastuen M, Lindblad M, Coleman EA, Ekstedt M. Measuring care transitions in Sweden: validation of the care transitions measure. International journal for quality in health care : journal of the International Society for Quality in Health Care / ISQua. 2018;30(4):291-7.
31. Osborne RH, Batterham RW, Elsworth GR, Hawkins M, Buchbinder R. The grounded psychometric development and initial validation of the Health Literacy Questionnaire (HLQ). BMC Public Health. 2013;13:658.
32. Chan AHY, Horne R, Hankins M, Chisari C. The Medication Adherence Report Scale: A measurement tool for eliciting patients' reports of nonadherence. Br J Clin Pharmacol. 2019.
33. Nasreddine ZS, Phillips NA, Bedirian V, Charbonneau S, Whitehead V, Collin I, et al. The Montreal Cognitive Assessment, MoCA: a brief screening tool for mild cognitive impairment. Journal of the American Geriatrics Society. 2005;53(4):695-9.
34. Zigmond AS, Snaith RP. The hospital anxiety and depression scale. Acta Psychiatr Scand. 1983;67(6):361-70.
35. Krupp LB, LaRocca NG, Muir-Nash J, Steinberg AD. The fatigue severity scale. Application to patients with multiple sclerosis and systemic lupus erythematosus. Arch Neurol. 1989;46(10):1121-3.
36. Duncan PW, Bode RK, Min Lai S, Perera S. Rasch analysis of a new stroke-specific outcome scale: the Stroke Impact Scale. Archives of physical medicine and rehabilitation. 2003;84(7):950-63.
37. Mahoney FI, Barthel DW. FUNCTIONAL EVALUATION: THE BARTHEL INDEX. Md State Med J. 1965;14:61-5.
38. Wade DT, Legh-Smith J, Langton Hewer R. Social activities after stroke: measurement and natural history using the Frenchay Activities Index. International rehabilitation medicine. 1985;7(4):176-81.

For peer review only

39. EuroQol--a new facility for the measurement of health-related quality of life. Health policy (Amsterdam, Netherlands). 1990;16(3):199-208.
40. Carlstedt E, Lexell EM, Pessah-Rasmussen H, Iwarsson S. Psychometric properties of the Swedish version of the General Self-Efficacy Scale in stroke survivors. Int J Rehabil Res. 2015;38(4):333-7.
41. Elmstahl S, Malmberg B, Annerstedt L. Caregiver's burden of patients 3 years after stroke assessed by a novel caregiver burden scale. Archives of physical medicine and rehabilitation. 1996;77(2):177-82.
42. Graneheim UH, Lundman B. Qualitative content analysis in nursing research: concepts, procedures and measures to achieve trustworthiness. Nurse education today. 2004;24(2):105-12.
43. Bate P, Robert G. Experience-based design: from redesigning the system around the patient to co-designing services with the patient. Qual Saf Health Care. 2006;15(5):307-10.

Legends

Figure 1, The co-designed care transition support

For peer review only - http://bmjopen.bmj.com/site/about/guidelines.xhtml

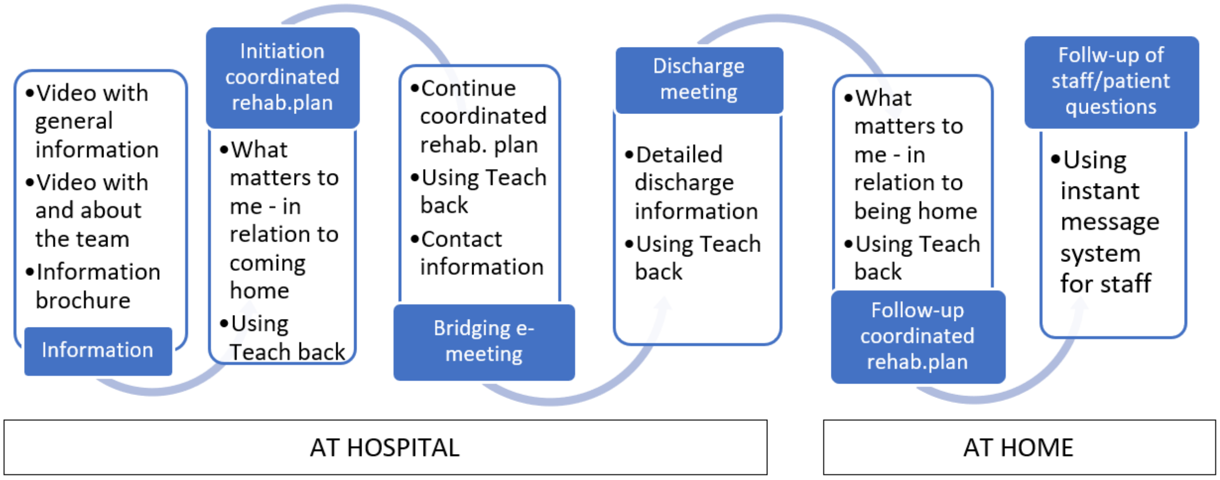

For peer review only

SPIRIT
STANDARD PROTOCOL ITEMS: RECOMMENDATIONS FOR INTERVENTIONAL TRIALS

SPIRIT 2013 Checklist: Recommended items to address in a clinical trial protocol and related documents*

**Section/item**	**ItemNo**	**Description**	
**Administrative information**			
Title	1	Descriptive title identifying the study design, population, interventions, and, if applicable, trial acronym	Title, page 1
Trial registration	2a	Trial identifier and registry name. If not yet registered, name of intended registry	Abstract, Page 2
	2b	All items from the World Health Organization Trial Registration Data Set	N/A
Protocol version	3	Date and version identifier	Abstract, Page 2
Funding	4	Sources and types of financial, material, and other support	Funding, Page 15
Roles and responsibilities	5a	Names, affiliations, and roles of protocol contributors	Title page, Page 1<br>Authors' contributions, page 15
	5b	Name and contact information for the trial sponsor	N/A
	5c	Role of study sponsor and funders, if any, in study design; collection, management, analysis, and interpretation of data; writing of the report; and the decision to submit the report for publication, including whether they will have ultimate authority over any of these activities	Funding, Page 15

For peer review only

For peer review only - http://bmjopen.bmj.com/site/about/guidelines.xhtml

	5d	Composition, oles, and responsibilities of the coordinating centre, steering committee, endpoint adjudication committee, data management team, and other individuals or groups overseeing the trial, if applicable (see Item 21a for data monitoring committee)	N/A
**Introduction**			
Background and rationale	6a	Description of research question and justification for undertaking the trial, including summary of relevant studies (published and unpublished) examining benefits and harms for each intervention	Background, Pages 3-5, Aims and hypothesis, page 9
	6b	Explanation for choice of comparators	Study design, Page 10
Objectives	7	Specific objectives or hypotheses	Aims and hypothesis, page 9
Trial design	8	Description of trial design including type of trial (eg, parallel group, crossover, factorial, single group), allocation ratio, and framework (eg, superiority, equivalence, noninferiority, exploratory)	Study design, Page 10
**Methods: Participants, interventions, and outcomes**			
Study setting	9	Description of study settings (eg, community clinic, academic hospital) and list of countries where data will be collected. Reference to where list of study sites can be obtained	Study design, Page 10
Eligibility criteria	10	Inclusion and exclusion criteria for participants. If applicable, eligibility criteria for study centres and individuals who will perform the interventions (eg, surgeons, psychotherapists)	Participants, Pages 10-11

For peer review only

Interventions	11a	Interventions for each group with sufficient detail to allow replication, including how and when they will be administered	Intervention Pages 6-8, Care transitions in control group, Page 11
	11b	Criteria for discontinuing or modifying allocated interventions for a given trial participant (eg, drug dose change in response to harms, participant request, or improving/worsening disease)	N/A
	11c	Strategies to improve adherence to intervention protocols, and any procedures for monitoring adherence (eg, drug tablet return, laboratory tests)	RQ1 and RQ2, Pages 11-12
	11d	Relevant concomitant care and interventions that are permitted or prohibited during the trial	N/A
Outcomes	12	Primary, secondary, and other outcomes, including the specific measurement variable (eg, systolic blood pressure), analysis metric (eg, change from baseline, final value, time to event), method of aggregation (eg, median, proportion), and time point for each outcome. Explanation of the clinical relevance of chosen efficacy and harm outcomes is strongly recommended	RQ3, Pages 12-13
Participant timeline	13	Time schedule of enrolment, interventions (including any run-ins and washouts), assessments, and visits for participants. A schematic diagram is highly recommended (see Figure)	Figure, Pages 6<br>RQ3, Page 13
Sample size	14	Estimated number of participants needed to achieve study objectives and how it was determined, including clinical and statistical assumptions supporting any sample size calculations	Patients and significant others, Page 10
Recruitment	15	Strategies for achieving adequate participant enrolment to reach target sample size	N/A
**Methods: Assignment of interventions (for controlled trials)**			
Allocation:			

Sequence generation	16a	Method of generating the allocation sequence (eg, computer-generated random numbers), and list of any factors for stratification. To reduce predictability of a random sequence, details of any planned restriction (eg, blocking) should be provided in a separate document that is unavailable to those who enrol participants or assign interventions	N/A
Allocation concealment mechanism	16b	Mechanism of implementing the allocation sequence (eg, central telephone; sequentially numbered, opaque, sealed envelopes), describing any steps to conceal the sequence until interventions are assigned	N/A
Implementation	16c	Who will generate the allocation sequence, who will enrol participants, and who will assign participants to interventions	N/A
Blinding (masking)	17a	Who will be blinded after assignment to interventions (eg, trial participants, care providers, outcome assessors, data analysts), and how	N/A
	17b	If blinded, circumstances under which unblinding is permissible, and procedure for revealing a participant's allocated intervention during the trial	N/A
**Methods: Data collection, management, and analysis**			
Data collection methods	18a	Plans for assessment and collection of outcome, baseline, and other trial data, including any related processes to promote data quality (eg, duplicate measurements, training of assessors) and a description of study instruments (eg, questionnaires, laboratory tests) along with their reliability and validity, if known. Reference to where data collection forms can be found, if not in the protocol	Data collection, Pages 11-13
	18b	Plans to promote participant retention and complete follow-up, including list of any outcome data to be collected for participants who discontinue or deviate from intervention protocols	N/A

Data management	19	Plans for data entry, coding, security, and storage, including any related processes to promote data quality (eg, double data entry; range checks for data values). Reference to where details of data management procedures can be found, if not in the protocol	N/A
Statistical methods	20a	Statistical methods for analysing primary and secondary outcomes. Reference to where other details of the statistical analysis plan can be found, if not in the protocol	Analyses, Page 13
	20b	Methods for any additional analyses (eg, subgroup and adjusted analyses)	N/A
	20c	Definition of analysis population relating to protocol non-adherence (eg, as randomised analysis), and any statistical methods to handle missing data (eg, multiple imputation)	N/A
**Methods: Monitoring**			
Data monitoring	21a	Composition of data monitoring committee (DMC); summary of its role and reporting structure; statement of whether it is independent from the sponsor and competing interests; and reference to where further details about its charter can be found, if not in the protocol. Alternatively, an explanation of why a DMC is not needed	N/A
	21b	Description of any interim analyses and stopping guidelines, including who will have access to these interim results and make the final decision to terminate the trial	N/A
Harms	22	Plans for collecting, assessing, reporting, and managing solicited and spontaneously reported adverse events and other unintended effects of trial interventions or trial conduct	N/A
Auditing	23	Frequency and procedures for auditing trial conduct, if any, and whether the process will be independent from investigators and the sponsor	N/A
**Ethics and dissemination**			

Research ethics approval	24	Plans for seeking research ethics committee/institutional review board (REC/IRB) approval	Ethics approval and consent to participate, Page 14-15
Protocol amendments	25	Plans for communicating important protocol modifications (eg, changes to eligibility criteria, outcomes, analyses) to relevant parties (eg, investigators, REC/IRBs, trial participants, trial registries, journals, regulators)	Methods, Page 9
Consent or assent	26a	Who will obtain informed consent or assent from potential trial participants or authorised surrogates, and how (see Item 32)	Patient and significant other, Page 10
	26b	Additional consent provisions for collection and use of participant data and biological specimens in ancillary studies, if applicable	N/A
Confidentiality	27	How personal information about potential and enrolled participants will be collected, shared, and maintained in order to protect confidentiality before, during, and after the trial	Ethics approval and consent to participate, Page 14-15
Declaration of interests	28	Financial and other competing interests for principal investigators for the overall trial and each study site	Funding, Page 15
Access to data	29	Statement of who will have access to the final trial dataset, and disclosure of contractual agreements that limit such access for investigators	Ethics approval and consent to participate, Page 14-15
Ancillary and post-trial care	30	Provisions, if any, for ancillary and post-trial care, and for compensation to those who suffer harm from trial participation	N/A
Dissemination policy	31a	Plans for investigators and sponsor to communicate trial results to participants, healthcare professionals, the public, and other relevant groups (eg, via publication, reporting in results databases, or other data sharing arrangements), including any publication restrictions	N/A
	31b	Authorship eligibility guidelines and any intended use of professional writers	N/A

For peer review only - http://bmjopen.bmj.com/site/about/guidelines.xhtml

	31c	Plans, if any, for granting public access to the full protocol, participant-level dataset, and statistical code	N/A
**Appendices**			
Informed consent materials	32	Model consent form and other related documentation given to participants and authorised surrogates	N/A
Biological specimens	33	Plans for collection, laboratory evaluation, and storage of biological specimens for genetic or molecular analysis in the current trial and for future use in ancillary studies, if applicable	N/A

*It is strongly recommended that this checklist be read in conjunction with the SPIRIT 2013 Explanation & Elaboration for important clarification on the items. Amendments to the protocol should be tracked and dated. The SPIRIT checklist is copyrighted by the SPIRIT Group under the Creative Commons "Attribution-NonCommercial-NoDerivs 3.0 Unported" license.

For peer review only

BMJ Open

Person-centred care transitions for people with stroke: Study protocol for a feasibility evaluation of co-designed care transition support

Journal:	*BMJ Open*
Manuscript ID	bmjopen-2020-047329.R2
Article Type:	Protocol
Date Submitted by the Author:	24-Sep-2021
Complete List of Authors:	Flink, Maria; Karolinska Institutet, Neurobiology, Care Sciences and Society; Karolinska University Hospital<br>Lindblom, Sebastian; Karolinska Institutet, Neurobiology, Care Sciences and Society; Karolinska University Hospital<br>Tistad, Malin; Dalarna University<br>Laska, Ann Charlotte ; Karolinska Institutet, Department of Clinical Sciences Danderyd Hospital<br>Bertilsson, Bo Christer; Karolinska Institutet, Neurobiology, Care Sciences and Society<br>Wärlinge, Carmen; Region Stockholm, Academic Primary Health Care Centre<br>Hasselström, Jan; Karolinska Institutet, 6. Department of Neurobiology, Care Sciences and Society; Region Stockholm, Academic Primary Health Care Centre<br>Elf, Marie; Dalarna University, School of education, health and social studies<br>von Koch, Lena; Karolinska Institutet, Neurobiology, Care Sciences and Society; Karolinska University Hospital<br>Ytterberg, Charlotte; Karolinska Institutet, Neurobiology, Care Sciences and Society; Karolinska University Hospital
**Primary Subject Heading**:	Neurology
Secondary Subject Heading:	Patient-centred medicine, Rehabilitation medicine
Keywords:	Stroke < NEUROLOGY, REHABILITATION MEDICINE, Organisation of health services < HEALTH SERVICES ADMINISTRATION & MANAGEMENT

SCHOLARONE™
Manuscripts

Person-centred care transitions for people with stroke: Study protocol for a feasibility evaluation of co-designed care transition support

Maria Flink[1,2], Sebastian Lindblom[1,2], Malin Tistad[1,3], Ann Charlotte Laska[4], Bo Christer Bertilsson[1], Carmen Wärlinge[5], Jan Hasselström[1,5], Marie Elf[3], Lena von Koch[1,2], Charlotte Ytterberg[1,2]

1. Karolinska Institutet, Department of Neurobiology, Care Sciences and Society, Huddinge, Sweden
2. Karolinska University Hospital, Stockholm, Sweden
3. Dalarna University, School of Education, Health and Social Studies, Falun, Sweden
4. Karolinska Institutet, Department of Clinical Sciences Danderyd Hospital, Stockholm, Sweden
5. Academic Primary Health Care Centre, Stockholm Health Care Services (SLSO), Region Stockholm, Sweden

Corresponding author:

Maria Flink

Alfred Nobels allé 23, D2

Karolinska Institutet

SE- 14183 Huddinge, SWEDEN

maria.flink@ki.se

Abstract

Background: Care transitions following stroke should be bridged with collaboration between hospital staff and home rehabilitation teams since well-coordinated transitions can reduce death and disability following a stroke. However, health services are delivered within

For peer review only

organisational structures, rather than being based on patients' needs. The aim of this study protocol is to assess the feasibility, operationalised here as fidelity and acceptability, of a co-designed care transition support for people with stroke.

Methods: This study protocol describes the evaluation of a feasibility study using a non-randomised controlled design. The co-designed care transition support includes patient information using videos, leaflets, and teach back; what-matters-to me dialogue; a coordinated rehabilitation plan; bridged e-meeting; and a message system for cross-organizational collaboration. Patients with stroke, first time or recurrent, who are to be discharged home from hospital and referred to a rehabilitation team in primary healthcare for continued rehabilitation in the home will be included. One week after stroke, data will be collected on the primary outcome, namely satisfaction with the care transition support, and on the secondary outcome, namely health literacy and medication adherence. Data on use of healthcare will be obtained from a register of healthcare contacts. The outcomes of patients and significant others will be compared with matched controls from other geriatric stroke and acute stroke units, and with matched historic controls from a previous dataset at the intervention and control units. Data on acceptability and fidelity will be assessed through interviews and observations at the intervention units.

Discussion: The care transition support addresses a poorly functioning part of care trajectories in current healthcare. The development of this co-designed care transition support has involved people with stroke, significant other, and healthcare professionals. Such involvement has the potential to better identify and re-conceptualise problems, and incorporate user experiences.

Trial registration: http://www.clinicaltrials.gov id: NCT02925871. Date of registration October 6, 2016

Protocol version: 1

Strengths and limitations of this study

- The intervention was developed following the guidelines of the Medical Research Council.
- The intervention was developed using a co-design methodology with people with stroke, significant other, and health care professionals.
- The use of mixed methods will provide knowledge regarding both process and outcomes
- The study is non-randomised, but the intervention group will be compared to matched controls

Key words

Stroke, care planning, continuity of patient care, implementation, participation, patient satisfaction, rehabilitation, self-efficacy, significant others, transitional care, user involvement

Background

Stroke, a leading causes of disability(1), has an abrupt onset and is a stressful event, both for persons with stroke, and their families(2). The past decades have seen improvements in medical and acute care of stroke as well as a reduction of the mean length of hospital stay in stroke units in high income countries(3). Strong evidence and consensus have shown that stroke care should initially be supplied in hospital stroke units in the acute phase(4), followed by a period of rehabilitation to regain functioning and to receive psychosocial support(2).

For peer review only

Thus, a care transition – a shift in responsibility from one healthcare setting to another –is almost always necessary. This may entail a care transition to inpatient rehabilitation, or directly to rehabilitation in the home provided by primary healthcare, e.g., a multi-professional team.

Uncoordinated care transitions impose a burden on patients and their significant others, especially when they lack information to navigate the healthcare system(2). The consequences of stroke such as aphasia, cognitive impairment, post-stroke fatigue, and depression(5), often render the stroke patients and their significant others unprepared for the care transition to the home(6, 7). This unpreparedness is further exacerbated by the sudden onset of stroke. In addition, a short hospital stay, little time to participate in transition planning(8, 9), and fragmented healthcare may contribute to patients' unrealistic expectations(7), and their sense of being abandoned(10) in a new and complex life situation. Although Swedish laws and other regulations require care providers to coordinate care, a great responsibility is put on patients and significant others to coordinate their own care(11, 12), while few resources are offered to support a patient's ability to participate in their own care or to self-manage(13, 14).

Several care transition interventions have been developed, both for people with stroke and for other populations. Strong evidence supports the conclusion that, to reduce re-hospitalisations, such care transitions must include both pre- and post-discharge activities, and support for patients' capacity for self-management(15, 16). Such pre- and post-discharge activities include discharge planning, structured discharge information, coordinated follow-up, and timely communication between care providers(17, 18). For people with stroke, calls have been made for self-management trials, as there is a lack of robust conclusions regarding

effectiveness (19). In stroke care transitions, extensive evidence shows that the rehabilitation care transition Early Supported Discharge (ESD) can reduce death and disability after a mild to moderate stroke(20). ESD implies that a stroke-specific, multidisciplinary team plans and coordinates hospital discharge, and provides rehabilitation in the community context(21). Despite the evidence in its favour, ESD has scarcely been implemented in Sweden. A possible explanation for the lack of implementation is that cross-organisational health services, like ESD, may be difficult to accomplish in fragmented healthcare systems with firm borders between hospital and community care.

There is hence a need for a rehabilitation care transition that consider patient's needs and capacities, as well as organisational settings. This need was highlighted in our prospective observational study, conducted between April 2016 and February 2018, in Stockholm, Sweden (22,23) with 206 people with acute stroke, of the current state of transitions from stroke and geriatric hospital units to the home with subsequent rehabilitation in primary healthcare. One week after hospital discharge, participants were followed-up regarding satisfaction with care transition; and then again, at three- and twelve-months, regarding functioning in everyday life and use of healthcare. In addition, we performed a grounded theory study of perceptions of current care transitions of patients, families, and healthcare professionals. The findings from the pre-studies(22, 23) identified a need for improved dialogue between patients and professionals; between professionals working in the same care provider organisation; and between professionals in different organisations. The improved dialogue means a communication where all parties communicate on equal terms and strive for a shared understanding. Further, a need to enhance patient preparedness for discharge, and self-management at home was identified. Based on the results(22, 23), we identified a need to develop bridging communicative links during the transition process, both within and between

For Peer review only

For peer review only - http://bmjopen.bmj.com/site/about/guidelines.xhtml

care provider organisations, as well as between patients and healthcare professionals. This links aims to ensure a coordinated rehabilitation transition, and to ensure that patients and their significant others to feel prepared for home coming.

Development of the intervention

The intervention is hence based both on our pre-studies(22, 23) and the stroke care trajectory literature(4, 20), and was developed in a two-step co-design process(24).

First, a trained facilitator held five co-design workshops with people with stroke, one significant other, and healthcare professionals from hospital stroke units and multidisciplinary home rehabilitation teams. Details of the co-design process and the participants have been presented previously (25). Three areas of unmet needs related to care transitions were identified across patients, significant others, and healthcare professionals:

1) Shared understanding of patient illness and situation. Participants identified a need for: repeated information about the present situation and expected future; communication adapted to the situation; updated written information; time to ask questions; structured and timely information.

2) Preparedness for homecoming. Participants identified a need for: feeling safe and understood at the discharge; clarity about services provided by the home rehabilitation team; preparedness for homecoming; clarity about medications; their perspectives and needs to be considered.

3) Coordination. Participants identified a need for: coordination between stroke unit and home rehabilitation teams; clarity about care trajectory; collaboration between sites.

Based on these needs, suggestions for improvements were co-designed by participants.

For peer review only

Thereafter, interactive e-meetings with healthcare professionals were held in which suggestions from the co-design workshops were further developed and contextually adapted into the intervention.

The intervention – the co-designed care transition support

The intervention consists of several elements that aim to meet patients', significant others', and professionals' needs for shared understanding, patient preparedness for homecoming, and coordination (Figure 1).

Please insert Figure about here.

Three different sources of home-rehabilitation information will be offered to patients and significant others: *two informational videos and one pamphlet*. First, an informational video including general information about home-rehabilitation will be shown during daytime in the day room. Second, an informational video featuring professionals from the patient's specific home-rehabilitation team will be provided to patients on digital tablets. Third, an informational pamphlet including general information about home-rehabilitation, a link to the information video, and contact information to the specific team, will be provided to patients. After the patient has accessed these sources of information, hospital staff will initiate a dialogue with the patient about '*what matters to me'* in relation to coming home and continued rehabilitation. In this dialogue, hospital staff and the patient will initate the development of a *coordinated rehabilitation plan* based on the patient's needs and wishes for continued rehabilitation at home, as well as hospital staffs' assessment. Based on agreements in the initiated plan, the hospital staff will refer the patient to the home-rehabilitation team and book an e-meeting with the team before discharge. This *bridged e-meeting* aims to connect the patient with the team that will be responsible for rehabilitation at home through a

For peer review only - http://bmjopen.bmj.com/site/about/guidelines.xhtml

first personal contact and decide an appointment for the first home-visit. The e-meetings will include a dialogue including the patient, the significant other, the hospital staff, and the rehabilitation team. The dialogue will include the patient's situation, needs, wishes, and fears in relation to coming home; a presentation from the home rehabilitation team about their services; a possibility for the patient and significant other to ask questions, and to continue the initiated coordinated rehabilitation plan for the homecoming. The e-meeting will function as an in-reaching bridge connecting patient, hospital staff, and home rehabilitation team. After homecoming, the home rehabilitation team, the patient, and significant others will review and revise the initiated coordinated rehabilitation plan.

Teach back(26, 27) is a person-centred, iterative communication method focused on a shared understanding of the patient's situation and the professional's information. The aim of *Teach back* is both to ensure that health professionals have understood the patient correctly, and to improve patient awareness of the own health condition, medications, and how to navigate the healthcare system, i.e., improved health literacy. Teach-back will be used in the *'what matters to me'* dialogue, the bridged e-meeting, and discharge encounters. The professionals will provide information and thereafter ask the patient repeat in their own words what was said, emphasising that this is to ensure that professionals explained satisfactorily. The detailed discharge information entails information about follow-up, what to consider after discharge, and where to turn with questions.

A safe *instant messaging system,* within the electronic health record, will ensure inter-organisational communication among healthcare professionals. This will ensure the exchange of information and resolve remaining questions regarding patient care between the hospital and home-rehabilitation, such as a revoked driving license or medications.

For peer review only - http://bmjopen.bmj.com/site/about/guidelines.xhtml

The co-designed care transition support is grounded in principals of person-centred care(28) and of integrated care(29) on a micro and meso level. This entails that the development of the intervention, and the intervention itself, are both based on individuals' preferences, needs, and values, taking patient resources into consideration, i.e., the intervention is person-centred(28), and strives to be realistic regarding what healthcare professionals in the included organisational settings can provide. We adhere to the person-centred 'Rainbow model of integrated care'(29). This model emphasises the need for both functional and normative links between patients and professionals, as well as within and between hospital and primary care organisations. Functional here refers to the information systems that link the hospital and primary care organisations. Normative here refers to shared understanding and values, both within and between organisations.

Care transitions in control group

Our previous studies identified that a rehabilitation care transition 'as usual' is initiated by an electronic referral from hospital healthcare professionals to the receiving neurorehabilitation team. The referral notifies the neurorehabilitation team about the patient and of the discharge. The neurorehabilitation team is obliged to initiate contact with the patient within 48 hours of referral and/or hospital discharge.

Aims and hypothesis

The primary aim is to assess the feasibility, operationalised as fidelity and acceptability, of a co-designed care transition support for people with stroke.

The following research questions (RQ) will be used:

RQ1: Is the intervention delivered as intended (fidelity)?

RQ2: Is the intervention acceptable in terms of content and delivery?

RQ3: Do patient and significant other outcomes differ between patients who received the intervention and matched controls, or between intervention patients and matched historic controls?

We hypothesise that information provided in a person-centred dialogue, as well as a discharge focused on bridging the gap between hospitalisation and home, will help patients and significant others feel more satisfied with the transition, and be better prepared for homecoming. This may, in turn, help patients regain functioning and reduce healthcare utilisation.

Methods

This study protocol describes the evaluation of feasibility for the co-designed care transition support using a non-randomised controlled design. Updates on the study protocol will be published on Clinical Trials.

The project adheres to the framework for development and evaluation of complex interventions by the Medical Research Council, UK(30), which calls for phased and iterative approaches in the design and evaluation of a complex intervention. This project hence draws upon our pre-studies of the perceptions, satisfaction, and outcomes of current state of care transitions, in which we have involved patients, significant others, and healthcare professionals in several care trajectories(22, 23).

Patient and public involvement

This study protocol for a feasibility study includes a description of the development of the intervention. The intervention was developed using a co-design methodology that involved

five workshops, followed by continuous discussions with people with stroke, a significant other, and healthcare professionals from hospital and primary care rehabilitation. The co-design included joint development of the interventions' components, contextual factors to consider, participant needs, and important outcomes to target. Details of the co-design process and the participants have been presented previously (25).

Study design

This is a feasibility study, with a non-randomised controlled study design. The intervention will be implemented at a geriatric stroke unit and an acute stroke unit in Hospital A in Stockholm, Sweden. The controls will be recruited from a geriatric stroke unit and an acute stroke unit at Hospital B in Stockholm, Sweden. Thereto, our previous studies at hospitals A and B (pre-study conducted from 2016-2018) provide historic controls using pre-data from the same hospital units. This design will allow us to compare intervention patients with matched controls, intervention patients with matched historic controls, and control patients with historic controls. Controls will be matched according to age, gender, stroke severity, and civil status (living alone or co-habiting).

Participants

Patients

Inclusion criteria: patients who have had a first time or recurrent stroke, and who will be discharged home from the participating stroke units and referred to a rehabilitation team in primary healthcare for continued rehabilitation in the home. Exclusion criteria: patients unable to give informed consent, due to e.g., severe aphasia (NIHSS (31)

language/communication 2 or 3) or dementia. Most patients discharged home from these units have mild to moderate stroke(28). The patients will be informed of the study and invited to participate at the hospital by a research assistant. The research assistant will provide oral and written information about the study and obtain consent to participate.

Sample size

In total, 50 persons will be consecutively included, 25 from the intervention site and 25 from the control site.

Significant others

Significant others will be included via the included patients. The included patients will be asked to name a significant other to be invited to also participate in the study. The significant others will be mailed written information about the study including an informed consent and a pre-stamped envelope. Significant others who return signed consent will be included in the study. Patients who do not have, or do not wish to name a significant other, will remain included.

Healthcare professionals

Hospital staff and home rehabilitation team members who have delivered the intervention will be invited to participate in the study. A purposive sampling (in terms of profession, years in profession) will be used. Approximately 15 hospital and home rehabilitation professionals will be included.

Data collection

RQ1 and RQ2

Data on acceptability and fidelity of the intervention will be collected by participant observations, interviews, and data from the healthcare record. At the intervention site, observations will be conducted of the ‘what matters to me’ dialogue, the bridged e-meeting, and discharge encounters. The observations will be made using a structured protocol in which the following criteria will be assessed: use of plain language, open-ended questions versus yes/no questions, asking patients to repeat information in their own words, repeating/revising information as needed. Individual interviews with the participating healthcare professionals and patients will be conducted after these observations. Interviews with healthcare professionals will target perspectives on patient understanding of information; the professionals' understanding of patient needs, resources, and will; experiences of collaboration with patients and healthcare professionals; and experiences of the ‘what matters to me’ dialogue, the bridged e-meeting, and discharge encounters. Patient interviews will target perspectives on their understanding of information material; how healthcare professionals explained information; experiences of collaboration with healthcare professionals; and how their perspectives and resources were considered.

After the 25 patients have been discharged with the co-designed care transition support, focus group interviews of healthcare professionals at the intervention site will be conducted regarding their overall understanding of the intervention (including the training), their experience delivering the intervention, and their perspectives on the content of the intervention. Patients and significant others at both the intervention and the control sites will be individually interviewed on their experiences of communication with healthcare professionals at hospital, how their needs were taken into consideration, and their satisfaction with and preparedness for discharge and homecoming. All interviews will be audio-recorded and transcribed verbatim.

Data on number of e-meetings and instant messages, and the content of the coordinated rehabilitation plan, will be collected from the healthcare records of the included patients.

RQ3

Data on likely effectiveness will be collected using questionnaires and registry data.

Baseline data on participant characteristics, length of hospital stay, disease-related data, and functioning in everyday life will be retrieved from medical records. Data on our primary outcome, satisfaction with co-designed care transition support, will be collected with the Care Transition Measure (32, 33). Data on our secondary outcome, health literacy, will be collected with the Health Literacy Questionnaire(34). These questionnaires together with an envelope (addressed and postage paid), will be sent by mail within one week of discharge from the hospital.

Data on the outcomes of patients and significant others at three months after stroke will be collected using questionnaires in structured interviews. Data will be collected from people with stroke regarding health literacy(34), medication adherence(35), cognitive function(36), signs of anxiety and depression(37), fatigue(38), perceived impact of stroke on function, activities and participation in everyday life(39), need of assistance in performing personal activities of daily living(40), participation in complex and social activities(41), health-related quality of life(42), and self-efficacy(43). Data on use of healthcare will be obtained from the register of healthcare contacts in the Region Stockholm. Data on caregiver burden(44), and informal care supplied will be collected using questionnaires in structured interviews with significant others. The same reliable and validated measures and data collection

For peer review only - http://bmjopen.bmj.com/site/about/guidelines.xhtml

timepoints have been applied in the pre-study of patient and significant other outcomes in current care transitions.

Analyses

Observations and interviews will be analysed using qualitative content analysis(45). Qualitative content analysis is a method well suited for descriptive qualitative analysis(45). Likely effectiveness of the co-designed care transition support (RQ3) in terms of outcomes and resource use, will be examined statistically and compared with control and pre-study historic data. The statistician performing this analysis will be blinded to group allocation.

Ethics and dissemination

Ethical approvals have been obtained from the Swedish Ethical Review Authority, nos. 2015/1923-31/2, 2019-04219, and 2021-02274. The proposed project will be conducted in accordance with the Declaration of Helsinki; oral and written consent to participate will be obtained from all participants. All study-related information about participants will be stored securely at the Karolinska Institutet. Only the researcher within the group will have access to data.

The results will be published open-access in peer-reviewed journals. Dissemination also includes presentation at national and international conferences.

For peer review only - http://bmjopen.bmj.com/site/about/guidelines.xhtml

Discussion

The primary aim is to assess the feasibility, operationalised as fidelity and acceptability, of the co-designed care transition support for people with stroke. The co-designed care transition support will address a poorly functioning part of the care trajectories in current healthcare, as identified in our pre-studies(22, 23) and the stroke care trajectory literature (4, 20). A recent Cochrane review identified that discharge planning studies mainly focused on individualised discharge plans in order to reduce readmissions(18). Such plans may render a small reduction in the number of hospital readmissions, but the development of care transitions has so far not involved patients or significant others, nor all the professionals among whom coordination is required. The development of this co-designed care transition support has therefore involved all these users (46). Such involvement has the potential to better identify and re-conceptualise problems and incorporate user experiences (46).

List of abbreviations

ESD - Early Supported Discharge

RQ – research question

Declaration

Consent to publish

Not applicable.

For peer review only - http://bmjopen.bmj.com/site/about/guidelines.xhtml

Availability of data and materials

Not applicable yet. This study protocol does not contain any data or materials that can be shared. Contact corresponding author for up-dated information on availability of data and materials.

Competing interests

The authors declare that they have no competing interests

Funding

This work was supported by the Doctoral School in Healthcare Sciences [2-134/2016], the Kamprad Family Foundation grant number [20190185], Neuro Sweden, and the Swedish Stroke Association. The funders neither had, nor will have, any role in design, data collection, data analysis, interpretation of data, writing of manuscripts or in decision to submit the paper for publication.

Authors' contributions

MF, SL, MT, ACL, ME, LvK, and CY drafted the manuscript. BCB, CW, and JH made significant contributions in revising and re-writing the manuscript. All authors have read and approved the manuscript.

Acknowledgements

Not applicable

For peer review only

References

1. Global, regional, and national burden of stroke, 1990-2016: a systematic analysis for the Global Burden of Disease Study 2016. Lancet Neurol. 2019;18(5):439-58.
2. Wissel J, Olver J, Sunnerhagen KS. Navigating the poststroke continuum of care. Journal of stroke and cerebrovascular diseases : the official journal of National Stroke Association. 2013;22(1):1-8.
3. Tistad M, Ytterberg C, Sjostrand C, Holmqvist LW, von Koch L. Shorter length of stay in the stroke unit: comparison between the 1990s and 2000s. Topics in stroke rehabilitation. 2012;19(2):172-81.
4. Langhorne P, Ramachandra S. Organised inpatient (stroke unit) care for stroke: network meta-analysis. Cochrane Database Syst Rev. 2020;4:Cd000197.
5. Aarnes R, Stubberud J, Lerdal A. A literature review of factors associated with fatigue after stroke and a proposal for a framework for clinical utility. Neuropsychological rehabilitation. 2019:1-28.
6. Connolly T, Mahoney E. Stroke survivors' experiences transitioning from hospital to home. J Clin Nurs. 2018;27(21-22):3979-87.
7. Wottrich AW, Astrom K, Lofgren M. On parallel tracks: newly home from hospital--people with stroke describe their expectations. Disability and rehabilitation. 2012;34(14):1218-24.
8. Almborg AH, Ulander K, Thulin A, Berg S. Patients' perceptions of their participation in discharge planning after acute stroke. Journal of Clinical Nursing. 2009;18(2):199-209.
9. Almborg AH, Ulander K, Thulin A, Berg S. Discharge planning of stroke patients: the relatives' perceptions of participation. J Clin Nurs. 18. England2009. p. 857-65.
10. Ytterberg C, von Koch L, Erikson A. Abandoned to the strains of daily life: a qualitative study of the long-term experiences in partners to persons after a mild to moderate stroke. Disability and rehabilitation. 2019;41(6):649-55.
11. (Vårdanalys) TSAfHaCSA. Patientlagen i praktiken – en baslinjemätning. 2015.
12. Gallacher K, Morrison D, Jani B, Macdonald S, May CR, Montori VM, et al. Uncovering treatment burden as a key concept for stroke care: a systematic review of qualitative research. PLoS medicine. 2013;10(6):e1001473.
13. Flink M, Ekstedt M. Planning for the Discharge, not for Patient Self-Management at Home - An Observational and Interview Study of Hospital Discharge. Int J Integr Care. 2017;17(6):1.
14. Gustafsson L, Bootle K. Client and carer experience of transition home from inpatient stroke rehabilitation. Disability and rehabilitation. 2013;35(16):1380-6.
15. Braet A, Weltens C, Sermeus W. Effectiveness of discharge interventions from hospital to home on hospital readmissions: a systematic review. JBI Database System Rev Implement Rep. 2016;14(2):106-73.
16. Leppin AL, Gionfriddo MR, Kessler M, Brito JP, Mair FS, Gallacher K, et al. Preventing 30-Day Hospital Readmissions: A Systematic Review and Meta-analysis of Randomized Trials. JAMA internal medicine. 2014.
17. Hesselink G, Schoonhoven L, Barach P, Spijker A, Gademan P, Kalkman C, et al. Improving patient handovers from hospital to primary care: a systematic review. Annals of internal medicine. 2012;157(6):417-28.
18. Goncalves-Bradley DC, Lannin NA, Clemson LM, Cameron ID, Shepperd S. Discharge planning from hospital. Cochrane Database Syst Rev. 2016(1):Cd000313.

For peer review only

19. Wray F, Clarke D, Forster A. Post-stroke self-management interventions: a systematic review of effectiveness and investigation of the inclusion of stroke survivors with aphasia. Disability and rehabilitation. 2018;40(11):1237-51.
20. Langhorne P, Baylan S. Early supported discharge services for people with acute stroke. Cochrane Database Syst Rev. 2017;7:Cd000443.
21. Fisher RJ, Gaynor C, Kerr M, Langhorne P, Anderson C, Bautz-Holter E, et al. A consensus on stroke: early supported discharge. Stroke; a journal of cerebral circulation. 2011;42(5):1392-7.
22. Lindblom S, Flink M, Sjöstrand S, Laska A, von Koch L, Ytterberg C. Perceived Quality of Care Transitions between Hospital and the Home in People with Stroke. J Am Med Dir Assoc. 2020.
23. Lindblom S, Ytterberg C, Elf M, Flink M. Perceptive Dialogue for Linking Stakeholders and Units During Care Transitions - A Qualitative Study of People with Stroke, Significant Others and Healthcare Professionals in Sweden. Int J Integr Care. 2020;20(1):11.
24. Boyd H, McKernon S, Mullin B, Old A. Improving healthcare through the use of co-design. N Z Med J. 2012;125(1357):76-87.
25. Lindblom S, Flink M, Elf M, Laska AC, von Koch L, Ytterberg C. The manifestation of participation within a co-design process involving patients, significant others and health-care professionals. Health Expect. 2021.
26. Ha Dinh TT, Bonner A, Clark R, Ramsbotham J, Hines S. The effectiveness of the teach-back method on adherence and self-management in health education for people with chronic disease: a systematic review. JBI Database System Rev Implement Rep. 2016;14(1):210-47.
27. Oh EG, Lee HJ, Yang YL, Kim YM. Effectiveness of Discharge Education With the Teach-Back Method on 30-Day Readmission: A Systematic Review. J Patient Saf. 2019.
28. Institute_of_Medicine. Crossing the quality chasm: a new health system for the twenty-first century. Washington: National Academies Press; 2001.
29. Valentijn PP. Rainbow of Chaos: A study into the Theory and Practice of Integrated Primary Care: Pim P. Valentijn, [S.l.: s.n.], 2015 (Print Service Ede), pp. 195, Doctoral Thesis Tilburg University, The Netherlands, ISBN: 978-94-91602-40-5. Int J Integr Care. 2016;16(2):3.
30. Craig P, Dieppe P, Macintyre S, Michie S, Nazareth I, Petticrew M. Developing and evaluating complex interventions: the new Medical Research Council guidance. Bmj. 2008;337:a1655.
31. Brott T, Adams HP, Jr., Olinger CP, Marler JR, Barsan WG, Biller J, et al. Measurements of acute cerebral infarction: a clinical examination scale. Stroke; a journal of cerebral circulation. 1989;20(7):864-70.
32. Coleman EA, Smith JD, Frank JC, Eilertsen TB, Thiare JN, Kramer AM. Development and testing of a measure designed to assess the quality of care transitions. Int J Integr Care. 2002;2:e02.
33. Flink M, Tessma M, Cvancarova Smastuen M, Lindblad M, Coleman EA, Ekstedt M. Measuring care transitions in Sweden: validation of the care transitions measure. International journal for quality in health care : journal of the International Society for Quality in Health Care / ISQua. 2018;30(4):291-7.
34. Osborne RH, Batterham RW, Elsworth GR, Hawkins M, Buchbinder R. The grounded psychometric development and initial validation of the Health Literacy Questionnaire (HLQ). BMC Public Health. 2013;13:658.

For peer review only - http://bmjopen.bmj.com/site/about/guidelines.xhtml

35. Chan AHY, Horne R, Hankins M, Chisari C. The Medication Adherence Report Scale: A measurement tool for eliciting patients' reports of nonadherence. Br J Clin Pharmacol. 2019.
36. Nasreddine ZS, Phillips NA, Bedirian V, Charbonneau S, Whitehead V, Collin I, et al. The Montreal Cognitive Assessment, MoCA: a brief screening tool for mild cognitive impairment. Journal of the American Geriatrics Society. 2005;53(4):695-9.
37. Zigmond AS, Snaith RP. The hospital anxiety and depression scale. Acta Psychiatr Scand. 1983;67(6):361-70.
38. Krupp LB, LaRocca NG, Muir-Nash J, Steinberg AD. The fatigue severity scale. Application to patients with multiple sclerosis and systemic lupus erythematosus. Arch Neurol. 1989;46(10):1121-3.
39. Duncan PW, Bode RK, Min Lai S, Perera S. Rasch analysis of a new stroke-specific outcome scale: the Stroke Impact Scale. Archives of physical medicine and rehabilitation. 2003;84(7):950-63.
40. Mahoney FI, Barthel DW. FUNCTIONAL EVALUATION: THE BARTHEL INDEX. Md State Med J. 1965;14:61-5.
41. Wade DT, Legh-Smith J, Langton Hewer R. Social activities after stroke: measurement and natural history using the Frenchay Activities Index. International rehabilitation medicine. 1985;7(4):176-81.
42. EuroQol--a new facility for the measurement of health-related quality of life. Health policy (Amsterdam, Netherlands). 1990;16(3):199-208.
43. Carlstedt E, Lexell EM, Pessah-Rasmussen H, Iwarsson S. Psychometric properties of the Swedish version of the General Self-Efficacy Scale in stroke survivors. Int J Rehabil Res. 2015;38(4):333-7.
44. Elmstahl S, Malmberg B, Annerstedt L. Caregiver's burden of patients 3 years after stroke assessed by a novel caregiver burden scale. Archives of physical medicine and rehabilitation. 1996;77(2):177-82.
45. Graneheim UH, Lundman B. Qualitative content analysis in nursing research: concepts, procedures and measures to achieve trustworthiness. Nurse education today. 2004;24(2):105-12.
46. Bate P, Robert G. Experience-based design: from redesigning the system around the patient to co-designing services with the patient. Qual Saf Health Care. 2006;15(5):307-10.

For peer review only

Legends

Figure 1, The co-designed care transition support

For peer review only - http://bmjopen.bmj.com/site/about/guidelines.xhtml

1

For peer review only

For peer review only - http://bmjopen.bmj.com/site/about/guidelines.xhtml

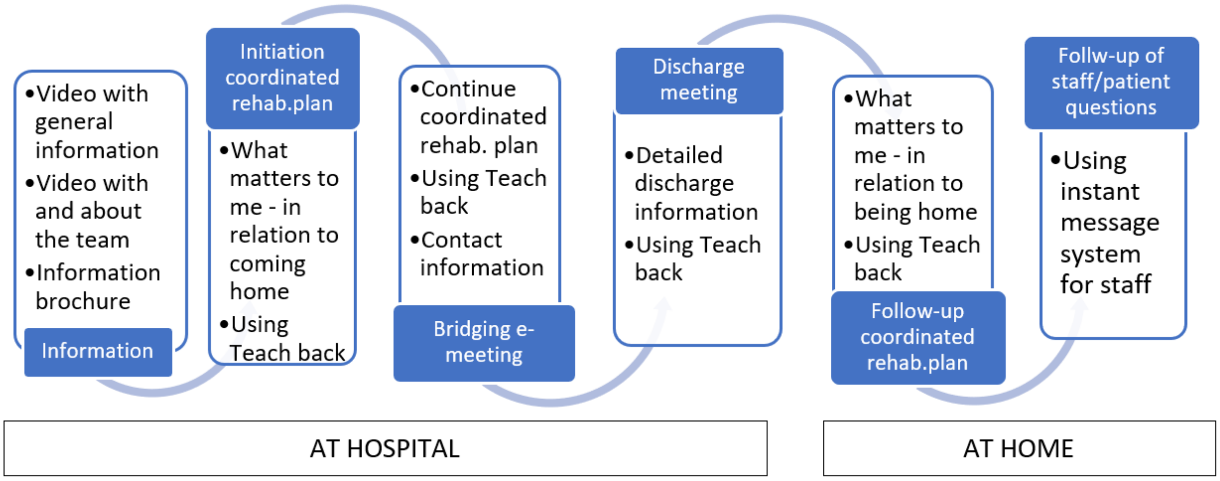

For peer review only

SPIRIT

STANDARD PROTOCOL ITEMS: RECOMMENDATIONS FOR INTERVENTIONAL TRIALS

SPIRIT 2013 Checklist: Recommended items to address in a clinical trial protocol and related documents*

**Section/item**	**ItemNo**	**Description**	
**Administrative information**			
Title	1	Descriptive title identifying the study design, population, interventions, and, if applicable, trial acronym	Title, page 1
Trial registration	2a	Trial identifier and registry name. If not yet registered, name of intended registry	Abstract, Page 2
	2b	All items from the World Health Organization Trial Registration Data Set	N/A
Protocol version	3	Date and version identifier	Abstract, Page 2
Funding	4	Sources and types of financial, material, and other support	Funding, Page 15
Roles and responsibilities	5a	Names, affiliations, and roles of protocol contributors	Title page, Page 1 Authors' contributions, page 15

For peer review only

	5b	Name and contact information for the trial sponsor	N/A
	5c	Role of study sponsor and funders, if any, in study design; collection, management, analysis, and interpretation of data; writing of the report; and the decision to submit the report for publication, including whether they will have ultimate authority over any of these activities	Funding, Page 15
	5d	Composition, oles, and responsibilities of the coordinating centre, steering committee, endpoint adjudication committee, data management team, and other individuals or groups overseeing the trial, if applicable (see Item 21a for data monitoring committee)	N/A
**Introduction**			
Background and rationale	6a	Description of research question and justification for undertaking the trial, including summary of relevant studies (published and unpublished) examining benefits and harms for each intervention	Background, Pages 3-5, Aims and hypothesis, page 9
	6b	Explanation for choice of comparators	Study design, Page 10
Objectives	7	Specific objectives or hypotheses	Aims and hypothesis, page 9
Trial design	8	Description of trial design including type of trial (eg, parallel group, crossover, factorial, single group), allocation ratio, and framework (eg, superiority, equivalence, noninferiority, exploratory)	Study design, Page 10

For peer review only

Methods: Participants, interventions, and outcomes			
Study setting	9	Description of study settings (eg, community clinic, academic hospital) and list of countries where data will be collected. Reference to where list of study sites can be obtained	Study design, Page 10
Eligibility criteria	10	Inclusion and exclusion criteria for participants. If applicable, eligibility criteria for study centres and individuals who will perform the interventions (eg, surgeons, psychotherapists)	Participants, Pages 10-11
Interventions	11a	Interventions for each group with sufficient detail to allow replication, including how and when they will be administered	Intervention Pages 6-8, Care transitions in control group, Page 11
	11b	Criteria for discontinuing or modifying allocated interventions for a given trial participant (eg, drug dose change in response to harms, participant request, or improving/worsening disease)	N/A
	11c	Strategies to improve adherence to intervention protocols, and any procedures for monitoring adherence (eg, drug tablet return, laboratory tests)	RQ1 and RQ2, Pages 11-12
	11d	Relevant concomitant care and interventions that are permitted or prohibited during the trial	N/A

Outcomes	12	Primary, secondary, and other outcomes, including the specific measurement variable (eg, systolic blood pressure), analysis metric (eg, change from baseline, final value, time to event), method of aggregation (eg, median, proportion), and time point for each outcome. Explanation of the clinical relevance of chosen efficacy and harm outcomes is strongly recommended	RQ3, Pages 12-13
Participant timeline	13	Time schedule of enrolment, interventions (including any run-ins and washouts), assessments, and visits for participants. A schematic diagram is highly recommended (see Figure)	Figure, Pages 6<br>RQ3, Page 13
Sample size	14	Estimated number of participants needed to achieve study objectives and how it was determined, including clinical and statistical assumptions supporting any sample size calculations	Patients and significant others,<br>Page 10
Recruitment	15	Strategies for achieving adequate participant enrolment to reach target sample size	N/A
**Methods: Assignment of interventions (for controlled trials)**			
Allocation:			
Sequence generation	16a	Method of generating the allocation sequence (eg, computer-generated random numbers), and list of any factors for stratification. To reduce predictability of a random sequence, details of any planned restriction (eg, blocking) should be provided in a separate document that is unavailable to those who enrol participants or assign interventions	N/A

Allocation concealment mechanism	16b	Mechanism of implementing the allocation sequence (eg, central telephone; sequentially numbered, opaque, sealed envelopes), describing any steps to conceal the sequence until interventions are assigned	N/A
Implementation	16c	Who will generate the allocation sequence, who will enrol participants, and who will assign participants to interventions	N/A
Blinding (masking)	17a	Who will be blinded after assignment to interventions (eg, trial participants, care providers, outcome assessors, data analysts), and how	N/A
	17b	If blinded, circumstances under which unblinding is permissible, and procedure for revealing a participant's allocated intervention during the trial	N/A
**Methods: Data collection, management, and analysis**			

Data collection methods	18a	Plans for assessment and collection of outcome, baseline, and other trial data, including any related processes to promote data quality (eg, duplicate measurements, training of assessors) and a description of study instruments (eg, questionnaires, laboratory tests) along with their reliability and validity, if known. Reference to where data collection forms can be found, if not in the protocol	Data collection, Pages 11-13
	18b	Plans to promote participant retention and complete follow-up, including list of any outcome data to be collected for participants who discontinue or deviate from intervention protocols	N/A
Data management	19	Plans for data entry, coding, security, and storage, including any related processes to promote data quality (eg, double data entry; range checks for data values). Reference to where details of data management procedures can be found, if not in the protocol	N/A
Statistical methods	20a	Statistical methods for analysing primary and secondary outcomes. Reference to where other details of the statistical analysis plan can be found, if not in the protocol	Analyses, Page 13
	20b	Methods for any additional analyses (eg, subgroup and adjusted analyses)	N/A
	20c	Definition of analysis population relating to protocol non-adherence (eg, as randomised analysis), and any statistical methods to handle missing data (eg, multiple imputation)	N/A
**Methods: Monitoring**			
Data monitoring	21a	Composition of data monitoring committee (DMC); summary of its role and reporting structure; statement of whether it is independent from the sponsor and competing interests; and reference to where further details about its charter can be found, if not in the protocol. Alternatively, an explanation of why a DMC is not needed	N/A

	21b	Description of any interim analyses and stopping guidelines, including who will have access to these interim results and make the final decision to terminate the trial	N/A
Harms	22	Plans for collecting, assessing, reporting, and managing solicited and spontaneously reported adverse events and other unintended effects of trial interventions or trial conduct	N/A
Auditin g	23	Frequency and procedures for auditing trial conduct, if any, and whether the process will be independent from investigators and the sponsor	N/A
**Ethics and dissemination**			
Resea rch ethics approv al	24	Plans for seeking research ethics committee/institutional review board (REC/IRB) approval	Ethics approval and consent to participate, Page 14-15
Protoc ol amend ments	25	Plans for communicating important protocol modifications (eg, changes to eligibility criteria, outcomes, analyses) to relevant parties (eg, investigators, REC/IRBs, trial participants, trial registries, journals, regulators)	Methods, Page 9
Conse nt or assent	26a	Who will obtain informed consent or assent from potential trial participants or authorised surrogates, and how (see Item 32)	Patient and significant other, Page 10
	26b	Additional consent provisions for collection and use of participant data and biological specimens in ancillary studies, if applicable	N/A

For peer review only

For peer review only - http://bmjopen.bmj.com/site/about/guidelines.xhtml

Confidentiality	27	How personal information about potential and enrolled participants will be collected, shared, and maintained in order to protect confidentiality before, during, and after the trial	Ethics approval and consent to participate, Page 14-15
Declaration of interests	28	Financial and other competing interests for principal investigators for the overall trial and each study site	Funding, Page 15
Access to data	29	Statement of who will have access to the final trial dataset, and disclosure of contractual agreements that limit such access for investigators	Ethics approval and consent to participate, Page 14-15
Ancillary and post-trial care	30	Provisions, if any, for ancillary and post-trial care, and for compensation to those who suffer harm from trial participation	N/A
Dissemination policy	31a	Plans for investigators and sponsor to communicate trial results to participants, healthcare professionals, the public, and other relevant groups (eg, via publication, reporting in results databases, or other data sharing arrangements), including any publication restrictions	N/A
	31b	Authorship eligibility guidelines and any intended use of professional writers	N/A

For peer review only - http://bmjopen.bmj.com/site/about/guidelines.xhtml

	31c	Plans, if any, for granting public access to the full protocol, participant-level dataset, and statistical code	N/A
**Appendices**			
Informed consent materials	32	Model consent form and other related documentation given to participants and authorised surrogates	N/A
Biological specimens	33	Plans for collection, laboratory evaluation, and storage of biological specimens for genetic or molecular analysis in the current trial and for future use in ancillary studies, if applicable	N/A

*It is strongly recommended that this checklist be read in conjunction with the SPIRIT 2013 Explanation & Elaboration for important clarification on the items. Amendments to the protocol should be tracked and dated. The SPIRIT checklist is copyrighted by the SPIRIT Group under the Creative Commons "Attribution-NonCommercial-NoDerivs 3.0 Unported" license.

For peer review only
